# Degradation of 2,4-dinitrotoluene in aqueous solution by dielectric barrier discharge plasma combined with Fe–RGO–BiVO_4_ nanocomposite

**DOI:** 10.1038/s41598-024-52286-y

**Published:** 2024-01-30

**Authors:** Yaser Vaziri, Ghorban Asgari, Farshid Ghorbani-Shahna, Tayyebeh Madrakian, Reza Shokoohi, Abdolmotaleb Seid-Mohammadi

**Affiliations:** 1 Department of Environmental Health Engineering, Hamadan University of Medical Science, Hamadan, Iran; 2 Social Determinants of Health Research Center (SDHRC), Faculty of Public Health, Department of Environmental Health Engineering, Hamadan University of Medical Sciences, Hamadan, Iran; 3 Center of Excellence for Occupational Health, Occupational Health and Safety Research Center, School of Public Health, Hamadan University of Medical Sciences, Hamadan, Iran; 4 Faculty of Chemistry, Bu-Ali Sina University, Hamadan, Iran

**Keywords:** Environmental sciences, Nanoscience and technology

## Abstract

2,4-Dinitrotoluene (2,4-DNT) as a priority and hazardous pollutant, is widely used in industrial and military activities. In this study the synergistic effect of Fe–RGO–BiVO_4_ nanocomposite in a non-thermal dielectric barrier discharge plasma reactor (NTP-DBD) for degrading 2,4-DNT was evaluated. Preparation of the Fe–RGO–BiVO_4_ nanocomposite was done by a stepwise chemical method depositing Fe and reduced graphene oxide (RGO) on BiVO_4_. Field emission scanning electron microscopy (FESEM), X-ray diffraction analysis (XRD), UV–vis diffuse reflectance spectra (DRS), and energy-dispersive X-ray spectroscopy mapping (EDS-mapping) validated the satisfactory synthesis of Fe–RGO–BiVO_4_. To find the optimal conditions and to determine the interaction of model parameters, a central composite design (RSM-CCD) had been employed. 2,4 DNT can be completely degraded at: initial 2,4-DNT concentration of 40 mg L^−1^, Fe–RGO–BiVO_4_ dosage of 0.75 g L^−1^, applied voltage of 21kV, reaction time of 30 min and pH equal to 7, while the single plasma process reached a degradation efficiency of 67%. The removal efficiency of chemical oxygen demand (COD) and total organic carbon (TOC) were 90.62% and 88.02% at 30 min contact time, respectively. Results also indicated that average oxidation state (AOS) and carbon oxidation state (COS) were enhanced in the catalytic NTP-DBD process, which demonstrate the effectiveness of proposed process for facilitating biodegradability of 2,4-DNT.

## Introduction

Increased industrial and military activities using nitro-aromatic explosives have led to a renewed focus on environmental effect of nitrated toluene compounds on surface water, groundwater, and soil^1,2^. DNTs mainly used as precursors to produce textile dyes, paints, synthetic leather, herbicides, fungicides, insecticides, polyurethane foam, explosives^1^ and car airbags^2^. Due to abundance, toxicity, mutagenicity and carcinogenicity of nitro-aromatic compounds, the US Environmental Protection Agency (USEPA) included dinitrotoluene isomers such as 2,4-DNT and 2,6-DNT in the list of priority pollutants for monitoring and control in aquatic environments^3^. DNT exposure poses serious risks to human health and can damage the reproductive system^1^. DNTs can enter the body through inhalation, skin contact, or ingestion of contaminated water^4^. There are so many studies have shown the adverse effects of 2,4-DNT on humans, plants and animals^5,6^. DNTs leak into the environment mainly through improper discharge of sewage and industrial waste. The presence of electron-inhibiting nitro groups makes conventional 2,4-DNT decomposition challenging^1^. Furthermore, DNT degrading organisms have been proven to have low cellular yields (biomass production) and produce little biomass^7^. Considering the mentioned problems, treatment of DNT-containing wastewater is vital to preserve human health and water bodies.

Currently, the most common method for treating DNT-contaminated wastewater is adsorption on activated carbon and incineration of resulted saturated carbon^8^. Adsorption methods could be efficiently used for the treatment of recalcitrant contaminants however these techniques are non-destructive and cause the movement of pollution from one phase to another, which may lead to the production of toxic secondary effluent contaminants.

Biological methods are not very popular in industry as a result of the presence of electron inhibitory nitro groups^5^, low cellular yield of DNTs degrading organisms (due to the presence of respiratory encoders and oxidative phosphorylation), long reaction time and high space required for this processes^9^. Among xenobiotics removal techniques, advanced oxidation processes (AOPs) have been highly considered to degrade non-biodegradable and refractory organic contaminants in aqueous environments.

The main goal of all AOP processes is to produce active and reactive species to decompose the target pollutants^10–14^. In these advanced processes, production of hydroxyl radicals (${{\text{OH}}}^{\cdot}$) is of particular importance due to its high oxidizing potential. Among the AOPs, photocatalytic processes using semiconductor compounds are recognized as reliable, low-cost and Eco-friendly techniques^10^. Due to the lower cost and ease practical application, researchers have paid much attention to find visible light photocatalysts^12^. Bismuth vanadate (BiVO_4_) as an emerging compound has attracted a lot of attention due to its outstanding properties, including low band gap, good dispensability, non-toxicity, optical corrosion resistance and remarkable photocatalytic efficiency in the decomposition of organic pollutants under visible light^12–16^. Studies on the monoclinic BiVO_4_ (m-BiVO_4_) have shown that its band gap energy is 2.4EV, which can be activated under visible light, and as a visible-light absorbing photocatalyst it can be used for organic pollutants degradation and wastewater treatment^12^. In pure BiVO_4_, the electric charges generated by visible light do not provide enough mobility for the electron–hole pair to properly separate them from the ground state, which is undesirable for the activity of a photocatalyst^13^. Appropriate modifications of pure BiVO_4_ i.e. heterojunction construction, ions doping, morphologies control, loading noble metal doping and the use of interface effect can improve the separation of electric charge carriers. The use of reduced graphene oxide (RGO) as a support substrate for BiVO_4_ is a suitable method due to its unique 2D carbon structure, excellent electrical conductivity and high surface area^14^. RGO not only limits the recombination of produced electron–hole pairs as an excellent electron receptor, but also absorbs large volumes of contaminants on the surface of the catalyst, making it a perfect co-catalyst to use in conjunction with semiconductor photocatalysts^15–17^. Moreover, doping small amounts of Fe on the photocatalyst increases the separation tendency of electron–hole pair, improves light absorption capacity, and consequently improves photocatalytic activity. For example, Regmi et al. obtained a higher efficiency in the removal of ibuprofen by doping Fe on the BiVO_4_ photocatalyst^18^. The BiVO_4_ photocatalyst modified by this method can overcome the common deficiencies of each component and increase the efficiency of the photocatalyst. Despite the advantages mentioned for BiVO_4_, most BiVO_4_-based catalysts have a relatively low efficiency (less than 80%) in less than 1 h and require more time (more than 2 h) to achieve efficiencies above 90%^19^. Consequently, one viable solution to the aforementioned problem is to combine other techniques with BiVO_4_-based catalysts to increase the rate and efficiency of organic pollutants removal.

Recently, Non-thermal plasma (NTP) has been widely used as an AOP method for the degradation of organic compounds in aquatic solution^20–22^. Water and wastewater treatment with non-thermal plasma technique does not require the addition of external chemical oxidants, temperature increase and pH adjustment^21,22^ and it is also possible to reuse the treated effluent. Other advantages of NTP include ease of operation, high reaction speed at room temperature and short residence time^20^. DBD plasma technology is a promising NTP method for wastewater treatment due to production of more uniform (homogenous) discharge, generation of more active oxidizing species, and having less electrode loss comparing to the gas corona discharge^21–23^. In the plasma discharge process, a large number of active compounds such as ${\text{H}}_{2} {\text{O}}_{2}$, ${\text{O}}_{3}$, ${\text{OH}}^{ \cdot }$, ${\text{H}}^{ \cdot }$ and hydrated electrons (e^–^) can be produced^20^. Among these active species, ${\text{OH}}^{ \cdot }$ has a higher oxidation potential and can oxidize most pollutants, especially organic pollutants. Furthermore, molecular species such as ${\text{H}}_{2} {\text{O}}_{2}$ and ${\text{O}}_{3}$ produced in the plasma processes can also react together to generate powerful oxidizing radicals, such as ${\text{OH}}^{ \cdot }$^21^. In addition to generating active radicals, the NTP process can generate shock waves, creating supercritical conditions in the electrical discharge channel, creating vacuum bubbles (similar to the ultrasonic process)^20^, and radiation of ultraviolet (UV) and visible light^23^. The plasma process is also able to activate catalysts and increase their efficiency^20–22^. However, high energy consumption and the possibility of producing side pollutants are limitations of the plasma process^21^.

The ability of the non-thermal plasma process to produce visible and ultraviolet light and catalyst activation has led recent studies to use a combined plasma and photocatalyst process. This integrated process can eliminate the limitations of both methods and lead to increased reaction speed and reduced retention time. To the best of our knowledge, no research has been carried on the removal of DNT using Fe–RGO–BiVO_4_ as photocatalyst combined with DBD reactor in aqueous solutions. Hence, the objective of the current study was to synthesis and evaluation of Fe–RGO–BiVO_4_ as a novel nanocomposite to study the efficiency of the combined DBD/Fe–RGO–BiVO_4_ process for removing DNT from aqueous solutions.

## Materials and methods

### Materials and photocatalyst preparation

All reagents used in the present study were analytical grade and utilized directly out of the package without further treatments. All of the studies were conducted using deionized water (DI) (18.2 M.cm).

### Synthesis of BiVO_4_

BiVO_4_ was prepared in a typical homogeneous precipitation method according to a previously reported method with minor modifications^24,25^. 30 g of Bi (NO_3_)_3_.5H_2_O was dissolved in 320 mL of HNO_3_ (1 M) aqueous solution under stirring till the formation of a clear solution. Afterwards 7 g of NH_4_VO_3_ (equal number of moles with Bi (NO_3_)_3_^·^5H_2_O) was poured into the reaction vessel. After heating for 5 min and ultrasonication for 30 min a yellowish orange mixture was obtained. The mixture was heated to 80 °C and maintained for 24 h after the addition of 30 g of urea. The resulting precipitate was washed thoroughly multiple times with deionized water and ethanol before being dried in an oven for 12 h at 60 °C.

### Synthesis of Fe–RGO–BiVO_4_

Graphene Oxide (GO) was fabricated by modified Hummers method^26^. Briefly, 2 g of graphite powder mixed in a 500-mL flask containing 1.5 g of NaNO_3_ and 50 mL of concentrated H_2_SO_4_ (98%). To prepare the mixture for next step, it was kept in an ice bath while being stirred for two hours. After that, 7.10 g of KMnO4 was added to the suspension gradually over the course of two hours to maintain the temperature under 25 °C. Then, the temperature of the combination increased by about 35 °C. Subsequently, the suspension was diluted by 150 mL of ultrapure water and mixed at 90 °C for 1 h. The reaction was completed by adding 150 mL of ultrapure water and 20 mL of H_2_O_2_ (30 wt. %). Once a bright yellow color has been achieved, the resulted GO suspension had been centrifuged, and washed several times with a solution of HCl and ultrapure water (3 wt. %). The obtained sample was finally dried in a vacuum oven for 24 h at 60 °C. To synthesize the RGO-BiVO_4_ composites, 0.03 g of prepared GO dispersed in de-ionized water (50 mL) and sonicated for 3 h. After that, 3 g of freshly produced BiVO_4_ was added and mixed continually until it became homogeneous after 3 h. Subsequently, 5 mL of NH_3_.H_2_O and 15 mL of N_2_H_4_ were sequentially added. After placing the suspension in a water bath (79–81 °C) and stirring it the for 3 h, the obtained sample was centrifuged, rinsed with water and ethanol for several times and dried at 60 °C overnight. Fe–RGO–BiVO_4_ nanocomposites were synthesized by immobilization of Fe nanoparticles onto the surface of RGO-BiVO_4_ via chemical reduction method in the presence of NaBH_4_^12,25^. In the typical reaction, 5 g of as-prepared RGO-BiVO_4_ was dispersed in DI water through sonication and magnetic stirring for 2 h at room temperature under N_2_ atmosphere. Then 0.25 g of FeCl_3_·6H_2_O was added in the deoxygenated RGO-BiVO_4_ solution and mixed for 12 h to ensure the adsorption of ferric ions onto RGO-BiVO_4_ surface. 0.5M NaBH_4_ solution were then added to the mixture drop wisely for the conversion of Fe^3+^ to Fe^O^ at a temperature of 25 °C. The resulted composite suspension was centrifuged and washed several times with ultrapure water and ethanol. The obtained sample was finally dried in a flask with continuous flow of nitrogen gas for 12 h at 50 °C.

### Characterization of nanocomposites

In this research, a Rigaku Ultima IV device were used for XRD analysis of synthesized products with Cu-kα radiation (45 kV, 40mA, λ = 1.54Å, 25 °C) in 2θ with the region of 10–80° to specify the structure and crystal phase of nanocomposite. The surface morphology and size of Fe–RGO–BiVO_4_ particles were determined by a FESEM device (FEI Nova Nano SEM 450). In order to discover the elemental composition and distribution of the nanocomposite along with surface and near-surface density of the nanocomposite, EDX with elemental mapping were used (BRUKER Flash 6|10]. FTIR (Thermo Avatar) was used to determine the molecular fingerprint, profile of the composite, and chemical bonds within it. UV–vis diffuse reflectance spectroscopy (UV–vis DRS), performed on a UV-2550 Shimadzu device, was used to assess the optical characteristics of the produced nanocomposite. According to Tauc's plot, the band gap energy, which is known as a key determinant of photocatalytic activity, can be computed as follows (Eq. 1)^27,28^:

1 $$\alpha \left( {h\nu } \right) = A\left( {h\nu - E_{g} } \right)^{n/2}$$

where α is the absorption coefficient, h is the Planck constant, ν is the light frequency, Eg is the band gap energy and A is the represents a constant. A semiconductor's optical transition type determines n, which are 1 and 4 for direct and indirect band-gap semiconductors, respectively. Pure BiVO_4_ had an n value of 4, and its calculated energy band gap was around 2.39 eV.

### Sample preparation

A stock solution of 2,4-DNT (200 mg ${{\text{L}}}^{-1}$) was prepared with DI and then attenuated in required levels. According to experimental design, 2,4-DNT samples were mixed and treated by the DBD/Fe–RGO–BiVO_4_ degradation reaction. The experiments were carried out in complete darkness. Samples' pH levels were adjusted using 0.1 N NaOH and ${{\text{H}}}_{2}{{\text{SO}}}_{4}.$ After the DBD/Fe–RGO–BiVO_4_ reaction was complete, a suitable portion of reaction solution was pipetted, centrifuged, and filtered.

### Analysis and measurement methods

A reverse-phase high-performance liquid chromatography system (HPLC) (Agilent 1200 Infinity, CA, USA) fitted with an Agilent Eclipse Plus C18 column (5 μm, 4.6 × 250 mm) and the Agilent 1260 Infinity Diode Array Detector (G4212B) was utilized to specify and determine the concentration of 2,4-DNT at 254 nm wavelength. Methanol and ultra-pure water made up the mobile phase (30/70, v/v). The injection volume was 20 μL, and the flow rate was set to 1.0 mL min^−1^. The intermediates of 2,4-DNT degradation were identified by LC–MS using a Waters Alliance 2695 HPLC-Micromass Quattro micro–API Mass Spectrometer equipped with an Atlantis T3-C18 column (3µm, 2.1 × 100 mm) at laboratory temperature, with injection volume of 25 µL and flow rate of 0.2 mL min^−1^. The mobile phase was a mixture of 60% acetonitrile + 0.1% formic acid and 40% water + 0.1% formic acid. Mass spectra (MS) conditions were as follows: Mode: ESI + , Cone Volt: 30 V, Capillary Volt: 4 kV, Extractor: 2 V, RF Lens: 0.2 V, Gas nebulizer: N_2_ (grade 5), Flow gas: 200 Lh^−1^, Source temperature: 120 °C, Desolvation temperature: 300 °C. Hach pH meter (HQ430D, USA) was used to determine the pH of solutions. The COD Cell Test (Merck photometric 25–1500 mg L^−1^ Spectroquant®) method along with a visible light spectrophotometry (DR 6000, Hach spectrophotometer) was used to determine COD. The TOC was evaluated by the TOC analyzer (Elementar Analysen systeme GmbH, Germany). The efficiency and kinetics of 2,4-DNT degradation in the DBD/Fe–RGO–BiVO_4_ system was calculated by Eqs. (2) and (3), respectively. Where, η represents the 2,4-DNT removal efficiency (%), ${{\text{C}}}_{0}$ and ${\text{C}}_{{\text{t}}}$ show the 2,4-DNT concentration (mg L^−1^) at zero and t time of the reaction, respectively, ${\text{k}}_{{{\text{obs}}}}$ is the pseudo-first order rate constant (min^−1^), and t (min) is the reaction time^25^.

2 $$\eta = \left( {1 - \frac{{{\text{C}}_{{\text{t}}} }}{{{\text{C}}_{0} }}} \right) \times 100$$

3 $${\text{Ln}}\left( {\frac{{{\text{C}}_{{\text{t}}} }}{{{\text{C}}_{0} }}} \right) = - {\text{k}}_{{{\text{obs}}}} \times {\text{t}}$$

### Design of experiments and process optimization

RSM has been widely applied for the statistical analysis and mathematical modeling of a research topic (variable or response) in which an interest response is impacted by two or more independent factors^29^. CCD is one of the most widely used RSM approaches for investigating the linear, interaction, and quadratic effects of independent variables on system response^30^. According to literature and conducting preliminary studies based on the full factorial design (2^K^), the independent variables' type and range were selected^27,30^. In this study, a five-level full orthogonal central composite design (OCCD) with the following levels: (-α, − 1, 0, + 1, + α) was utilized to optimize and study the effect of five independent variables including reaction time, initial solution pH, 2,4-DNT concentration, Fe–RGO–BiVO_4_ concentration, and applied voltage. Table 1 provides an overview of the independent variables and their coded levels.

**Table 1:** The symbols of five independent variables and their levels (α = 2).

Variables	Unit	Symbols	Levels				
			− α	− 1	0	+ 1	+ α
2,4-DNT concentration	mg/L	A	20	40	60	80	100
Fe–RGO–BiVO_4_ concentration	g/L	B	0	0.25	0.5	0.75	1
Applied voltage	kV	C	12	16.5	21	25.5	30
Reaction time	Min	D	10	15	20	25	30
pH	-	E	3	5	7	9	11

The selection of α in accordance with Eq. (4) is a requirement for creating an OCCD^29^.

4 $$\alpha = \left( {\frac{{\sqrt {{\text{Nf}}} - {\text{f}}}}{2}} \right)^{1/2}$$

Here, N is the total number of experiments, while f is denotes factorial points. Equations (5) and (6) were respectively used to derive N and f.

5 $${\text{N}} = 2^{{\text{K}}} + 2{\text{K}} + {\text{C}}$$

6 $${\text{f}} = 2^{{\text{K}}}$$

where, the number of independent variables is indicated by K. As a result, a three-level full face centered CCD with five independent variables offers 50 trials (N)^29,30^. According to Eq. (3), the chosen α value was 2. Designed experiments were repeated three times to acquire more accurate results. Design-Expert 12.0 software was used for experimental design, data analysis, and final optimization.

### Experimental setup

**Figure 1:**
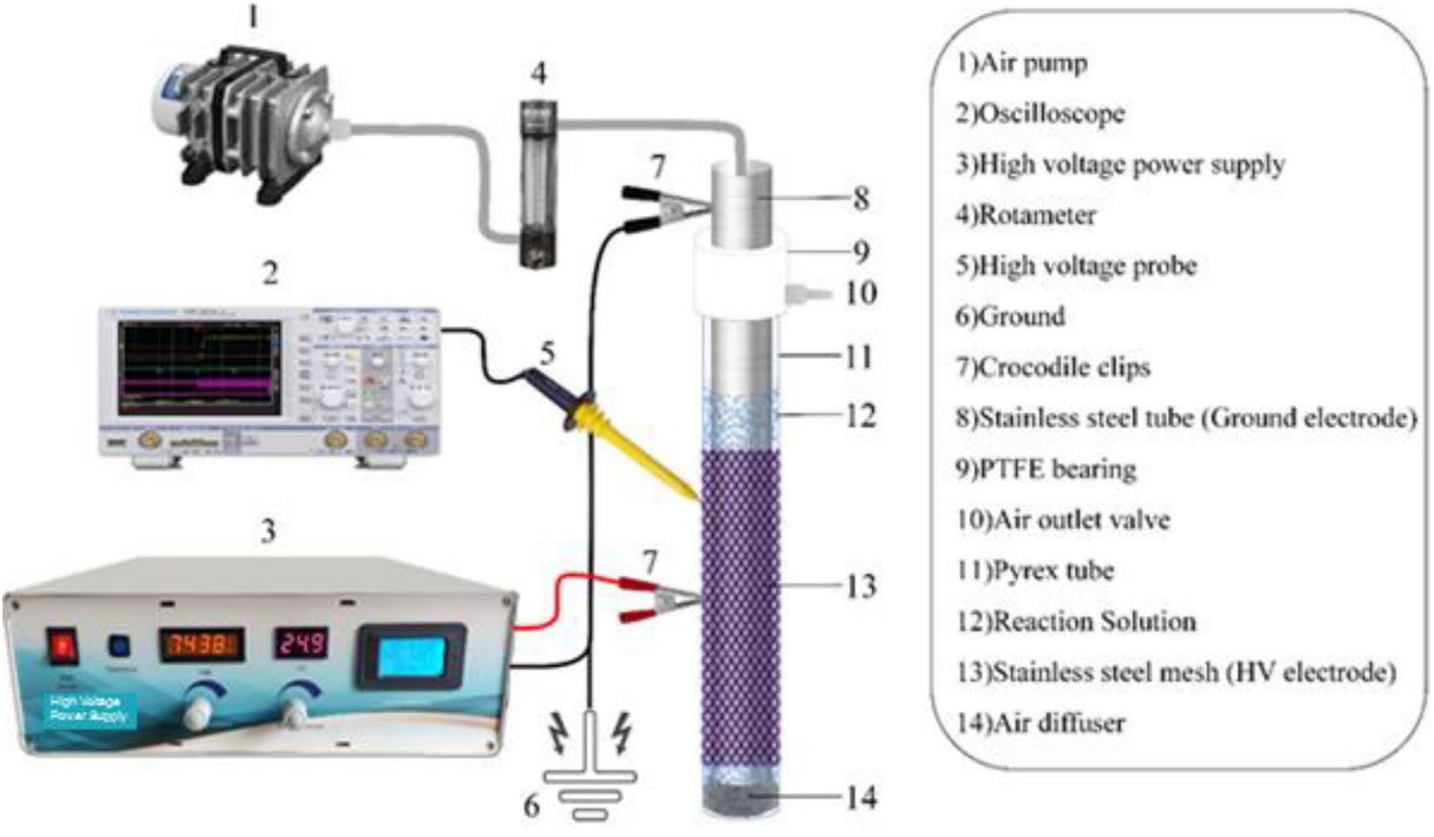
Schematic of the experimental setup for NTP- DBD reactor.

The experiments were carried out in a dark environment inside a vertical lab-scale DBD plasma reactor (Fig. 1). A Pyrex tube serving as the dielectric barrier was installed between the high voltage (HV) discharge electrode and ground electrode. The thickness, outer diameter, and height of the Pyrex tube were 1.2, 32 and, 500 mm, respectively. A stainless-steel mesh with a height of 300 mm wrapped over the Pyrex tube was the outer electrode and was connected to HV. The ground electrode was a stainless-steel tube with an outer diameter of 20 mm that was positioned in the cylindrical Pyrex’s center. Polytetrafluoroethylene (PTFE) bearings were used to keep the ground electrode at the center of the cylindrical Pyrex. The air diffuser was located at the bottom of the reactor below the ground electrode. A power meter (Instek, GPM-8213GP) was used to measure the input power. The applied voltage was monitored by a high voltage probe (Tektronix, P6015A) connected to a digital oscilloscope (Tektronix, DPO 3012). The parallel HV and ground electrodes had a 6 mm space between them. Prepared sample was filled in the internal gap distance between the inner surface of the dielectric Pyrex tube and the outer surface of ground electrode. The induced discharge and produced active species (e.g.,${{\text{H}}}_{2}{{\text{O}}}_{2}$, O_3_, etc.) were propagated throughout the sample during the operation of the plasma reactor. The high voltage for plasma formation was applied using an AC power supply (1–30 kV, 20 kHz, PSAC-1000, SATIA, Iran).

## Results and discussion

### Characterization of prepared catalysts

**Figure 2:**
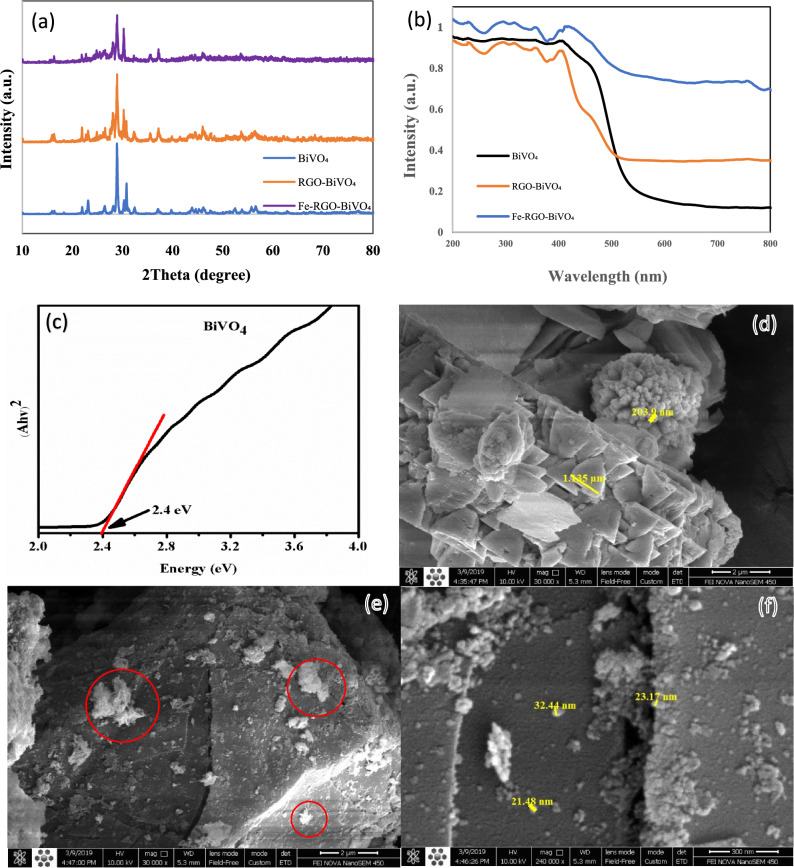
(**a**) XRD patterns; (**b**) UV–vis DRS spectra; (**c**) Tauc plot of pure and modified BiVO4; FESEM surface morphology images of (**d**) pure BiVO4, and (**e**, **f**) Fe–RGO–BiVO_4_.

Figure 2a presents the XRD patterns of the obtained photocatalysts which can reveal details about their crystalline structure. All sample patterns depicted distinct diffraction peaks, demonstrating the materials' strong crystallinity. Also, it could be concluded that all diffraction peaks observed for undoped BiVO_4_ could be indexed perfectly as monoclinic scheelite BiVO_4_ phase (JCPDS card No-14–0688)^31,32^. For RGO-BiVO_4_ and Fe–RGO–BiVO_4_, there were no identifiable diffraction peaks of iron species (44.7 and 65.07 for Fe (^o^), 33.22, 35.69 and 54.13 for Fe(II) and 24.5 and 32.3 for Fe(III)^27,28^ or RGO (2θ = 26° and 44°) for the modified BiVO_4_^33^. This could be attributed to the low diffraction intensity and low amount of metallic Fe and RGO in the related nanocomposites^34^. However, broader diffraction peaks of Fe–RGO–BiVO_4_ comparing to pure BiVO_4_ indicating the presence of smaller crystallites after iron loading^25^. Besides, iron loading has no effect on the crystal structure of BiVO_4_. It can be postulated that the loading of iron particles might be occurred exclusively on BiVO_4_ surface and was not anchored deep in the BiVO_4_ composition. It is reported that the number of photo-generated electrons and holes is directly affected spectral absorption^25^, so the UV–vis diffuse reflectance spectra (DRS) were used to examine the optical absorption characteristics of bare and modified BiVO_4_ samples. As presented in Fig. 2b, the pure BiVO_4_ (edge band = 2.4 eV) displayed an absorption edge at ca. 517 nm. The capacity of BiVO_4_ to absorb visible light was found to be improved in the presence of RGO and Fe on its surface. The maximum visible-light absorption edge was shown by Fe–RGO–BiVO_4_, which could be assigned to the SPR effect of metallic Fe and the grafting of RGO. The findings showed that the co-effect was a useful technique to improve the visible light performance of a single semiconductor. Besides, the corresponding energy band gap (Eg) for pure BiVO_4_ (calculated by the Eq. 1) was about 2.4 eV (Fig. 2c).

**Figure 3:**
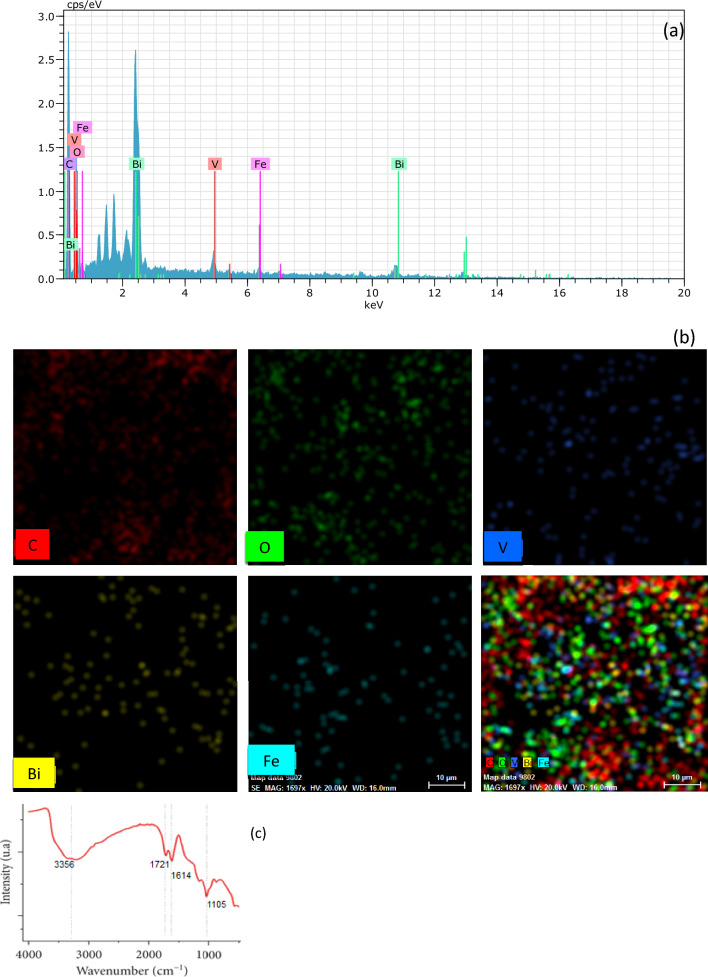
(**a**) EDX-Mapping spectra; (**b**) Elemental EDX of Fe–RGO–BiVO_4_, and (**c**) FTIR of GO.

The surface morphology of net BiVO_4_ and Fe–RGO–BiVO_4_ determined by FESEM images are shown in Fig. 2d, e and f. According to FESEM images, pure BiVO_4_ polyhedrons show a well-defined truncated bipyramid shape with highly active (040) facets exposed, and the surfaces are smooth (Fig. 2d). As shown in Fig. 2f, the surface of BiVO_4_ had uniformly distributed Fe particles^32^. Additionally, Fig. 2f demonstrates that the Fe particles are also spherical, with diameters ranging from 21 to 33 nm. Moreover, the FESEM observation of Fe–RGO–BiVO_4_ composites (Fig. 2e) displays stratiform RGO sheets (marked by red circles), despite the low dosages. It is apparent that the surface morphology of BiVO_4_ was not significantly affected by the deposition of Fe and RGO on the BiVO_4_ which supports findings from prior studies^33–35^. The EDX-Mapping spectra analysis of the Fe–RGO–BiVO_4_ is shown in Fig. 3a and b. As it stands, chemical composition of the catalyst consists of bismuth (40.21% wt.), carbon (30.68% wt.), oxygen (23.22% wt.), vanadium (4.52% wt.) and iron (1.38% wt.). Also, mapping analysis indicates the uniform distribution of C, Fe and O (Fe-RGO) on BiVO_4_ surface.

The FTIR spectrum of GO in Fig. 3c shows a broad peak appeared at 3356 cm^−1^ in the high frequency area attributed to the stretching mode of O–H bond, reveals the presence of hydroxyl groups in graphene oxide. The band observed at 1721 cm^−1^ was assigned to the carboxyl group. The sharp peak found at 1614 cm^−1^ is a resonance peak that can be assigned to the stretching and bending vibration of OH groups of water molecules adsorbed on graphene oxide. The peak at 1105 cm^−1^ corresponds to the vibrational mode of the C-O group.

### Data analysis and process modeling

Table 2 displays the complete CCD matrix along with the obtained experimental results (2,4-DNT removal rate). Table 2 shows the full CCD matrix with the experimental 2,4-DNT removal efficiency (obtained results). The *p *value and F-value calculated by ANOVA were applied to assess the suggested model's suitability for response prediction as well as the significant levels of the model and its related terms. Determination coefficients including R^2^ and adjusted R^2^ were used to evaluate the model Quality and accuracy^36^.

**Table 2:** CCD matrix with the experimental 2,4-DNT removal efficiency.

Run	Variables					Actual removal (%)	Predicted removal (%)	Run	Variables					Actual removal (%)	Predicted removal (%)
	A	B	C	D	E				A	B	C	D	E		
1	0	2	0	0	0	87.00	88.60	24	0	0	0	0	0	72.79	72.04
2	0	0	2	0	0	88.62	87.16	25	0	0	− 2	0	0	68.11	71.84
3	0	0	0	− 2	0	66.76	68.02	26	− 1	1	1	1	1	97.37	97.36
4	− 1	− 1	− 1	1	− 1	80.00	79.00	27	0	0	0	0	0	73.57	72.04
5	0	0	0	0	2	49.01	50.03	28	1	− 1	1	− 1	1	51.85	52.60
6	1	1	− 1	− 1	− 1	61.81	60.67	29	− 1	1	− 1	− 1	1	82.24	81.58
7	1	− 1	− 1	1	1	54.11	54.71	30	1	1	− 1	− 1	1	64.84	64.10
8	1	− 1	1	1	1	70.15	70.42	31	− 2	0	0	0	0	100.00	103.37
9	1	− 1	− 1	− 1	− 1	39.83	39.79	32	− 1	− 1	− 1	− 1	− 1	72.98	73.12
10	0	0	0	2	0	92.64	93.65	33	1	1	− 1	1	− 1	73.37	73.28
11	2	0	0	0	0	55.76	54.66	34	− 1	1	1	1	− 1	93.88	93.41
12	− 1	1	− 1	1	− 1	89.29	88.29	35	1	1	1	− 1	− 1	56.41	57.56
13	1	1	1	− 1	1	61.84	62.23	36	− 1	1	− 1	1	1	91.98	91.02
14	0	0	0	0	− 2	42.23	43.48	37	− 1	− 1	1	− 1	− 1	85.29	84.81
15	− 1	1	1	− 1	− 1	81.22	80.09	38	1	1	1	1	1	82.90	81.97
16	1	1	− 1	1	1	78.20	78.35	39	− 1	− 1	1	− 1	1	86.78	86.30
17	0	0	0	0	0	72.01	72.04	40	− 1	− 1	− 1	1	1	82.34	80.89
18	− 1	− 1	− 1	− 1	1	74.66	73.38	41	0	− 2	0	0	0	69.00	69.67
19	1	− 1	1	− 1	− 1	48.93	48.77	42	− 1	1	− 1	− 1	− 1	81.37	80.49
20	− 1	− 1	1	1	1	99.01	99.32	43	1	1	1	1	− 1	74.98	75.68
21	1	− 1	1	1	− 1	64.58	64.96	44	− 1	− 1	1	1	− 1	96.00	96.20
22	1	− 1	− 1	− 1	1	42.80	42.40	45	− 1	1	1	− 1	1	82.28	82.42
23	1	− 1	− 1	1	− 1	51.44	50.47								

Based on variations in the system's response to independent variables, a quadratic model was suggested as a reliable model to estimate the removal of 2,4-DNT in the DBD/Fe–RGO–BiVO_4_ process. By removing non-significant predictors based on the ANOVA analysis, the final derived model can provide a more accurate fitting to the data. Accordingly, by comparing the critical F-value (F_0.05_ (1, 26) = 4.35) with the observed F-values and the considering of *p *value = 0.05 as significant, a simplified polynomial quadratic model, that is provided in Eq. (7), was developed by excluding the non-significant predictors from the model. The suggested model is significant and well-fitted to the experimental data, as indicated by the model F-value of 247.58, which is significantly higher than the crucial value from Fisher's table (F_0.05_ (20, 24) = 2.03)^36^. This means that the probability of causing an F-value this large due to noise is extremely low (0.01%). Table 3 displays the remaining significant parameters of the model. The model's F-value and *p *value are 247.58 and < 0.0001, respectively, which prove that the model is significant. The R^2^ (determination coefficient) value, which is close to unity (0.9952), indicates that the derived regression models are well fitted. This means that 99.52% of the variability in the 2,4-DNT removal by DBD/Fe–RGO–BiVO_4_ process can be explained by the model. As manifested in Table 3, the slight difference between the R^2^ and R^2^_Adj_ implies that non-significant terms have a little chance to be included in the model^37^.

7 $$\begin{aligned}Removal\;Efficiency\;(\% ) & = 72.04 - 12.18(2,4 - DNT) + 4.73(Fe - RGO - BiVO4) + 3.83(voltage) \\ &\quad + 6.41(Time) + 1.64(pH) + 3.38(2,4 - DNT \times Fe - RGO - BiVO4) \\ &\quad - 0.6792(2,4 - DNT \times voltage) + 1.20(2,4 - DNT \times Time) \\ &\quad + 0.5852(2,4 - DNT \times pH) - 3.02(Fe - RGO - BiVO4 \times voltage) \\ &\quad + 0.4829(Fe - RGO - BiVO4 \times Time) + 0.2077(Fe - RGO - BiVO4 \times pH)\\ &\quad + 1.38(voltage \times Time) + 0.3072(voltage \times pH) + 0.4079(Time \times pH)\\ &\quad + 1.74(2,4 - DNT^{2} ) + 1.77(Fe - RGO - BiVO4^{2} ) + 1.87(voltage^{2} ) \\ &\quad + 2.2(Time^{2} ) - 6.32(pH^{2} ) \end{aligned}$$

**Table 3:** ANOVA model fitting results for the quadratic model.

Source	Sum of squares	df	Mean square	F-value	*p *value
Model	11,707.57	20	585.38	247.58	< 0.0001
A-DNT concentration	5932.40	1	5932.40	2509.08	< 0.0001
B-Nanocomposite Concentration	895.15	1	895.15	378.60	< 0.0001
C-Voltage	587.02	1	587.02	248.28	< 0.0001
D-Reaction time	1641.86	1	1641.86	694.42	< 0.0001
E-pH	107.40	1	107.40	45.42	< 0.0001
AB	365.01	1	365.01	154.38	< 0.0001
AC	14.76	1	14.76	6.24	0.0197
AD	46.07	1	46.07	19.49	0.0002
AE	10.96	1	10.96	4.64	0.0416
BC	292.08	1	292.08	123.53	< 0.0001
BD	7.46	1	7.46	3.16	0.0883
BE	1.38	1	1.38	0.5836	0.4524
CD	60.69	1	60.69	25.67	< 0.0001
CE	3.02	1	3.02	1.28	0.2696
DE	5.32	1	5.32	2.25	0.1465
A^2^	77.94	1	77.94	32.97	< 0.0001
B^2^	80.65	1	80.65	34.11	< 0.0001
C^2^	89.17	1	89.17	37.71	< 0.0001
D^2^	123.81	1	123.81	52.36	< 0.0001
E^2^	1022.55	1	1022.55	432.48	< 0.0001
Residual	56.74	24	2.36		
Lack of Fit	55.53	22	2.52	4.16	0.2116
Pure Error	1.21	2	0.6067		
Cor Total	11,764.31	44			

R^2^ = 0.9952, R^2^ adjusted = 0.9912, R^2^ predicted = 0.9806, Adequate Precision = 68.5261.

The Predicted R^2^ of 0.9806 is in acceptable agreement with the Adjusted R^2^ of 0.9912; because of the difference of less than 0.2. The regression model's reproducibility and variability in the mean value can both be described by the coefficient of variation (CV). The CV value (2.10) in this study was favorable given the acceptable range (0.5–13.5%), that affirms the reproducibility of the model^27^. The adequate precision statistically shows the signal-to-noise ratio, and values more than 4 are desirable for the model^35^. Since the signal to noise ratio of 60.5261achieved, the signal of the model is clearly adequate. In other words, the obtained model has the ability to predict and navigate the design space. Non-significant lack of fit is the requisite that shows the model is fitted. The *p *value (0.2116) and F-value (4.16) for lack of fit indicated that in comparison to the pure error, the model's lack of fit was not statistically significant (0.2116 > 0.05), and consequently, the model fits well with the available data^28^. In addition, there is only a 21.16% chance that noise will result in a lack of fit F-value this large.

**Figure 4:**
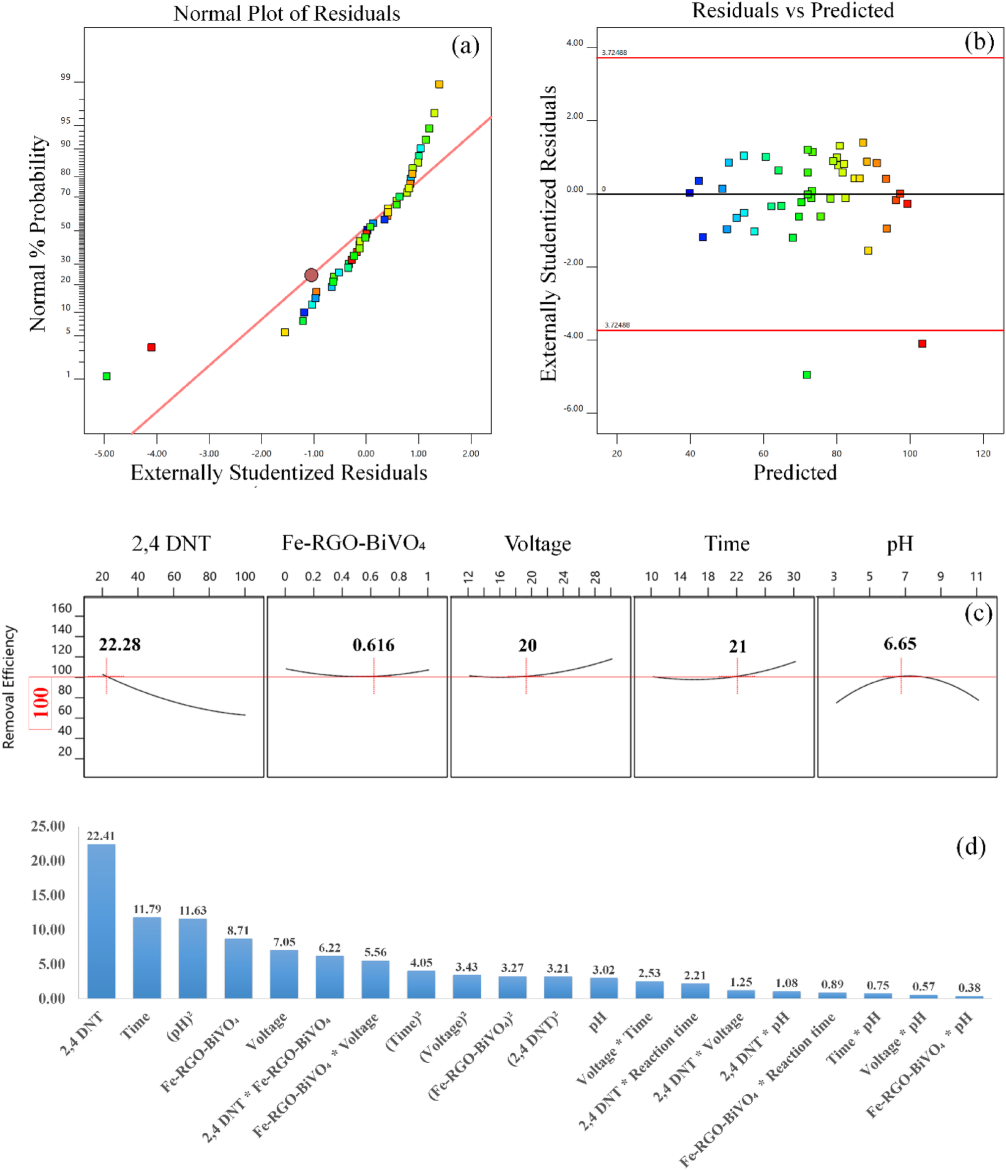
The diagnostic plots: (**a**) normal plot of residuals and (**b**) residuals versus predicted. (**c**) The optimal points obtained by CCD and (**d**) The Pareto effect of each term on 2.4-DNT removal.

Analyzing diagnostic plots is another way for evaluating the model's adequacy. Figure 4a and d provide the main diagnostic plots, such as the normal plot of residuals and residual versus predicted. The term residuals refer to the difference between the predicted and experimental values. Therefore, the normal probability plot of the residuals can display the normality of the data. As shown in the normal probability plot (Fig. 4a), all data points distributed near the middle straight line, suggesting.

that there is no need for response transformation because the residuals in the model prediction are normally distributed. All residuals are distributed randomly within the standard deviation range (3.72) without any outlier, as shown in Fig. 4b. It demonstrates that there is no need to repeat the experiments^38^. Eventually, considering the specified significance level, the proposed model's final equation in terms of actual factors is depicted in Eq. (7). The synergistic and antagonistic effects, respectively, are denoted by the positive and negative signs placed in front of the interaction terms.

### Effect of variables and process optimization

To predict the maximum 2,4-DNT removal efficiency achievable by the DBD/Fe–RGO–BiVO_4_ process, CCD method have been employed for process optimization. The "in range" mode was selected for five independent input variables and the model output (2,4-DNT removal efficiency) was set to "maximum" mode. Among the solutions provided by proposed model, first derivative solution presented the optimal conditions for DBD/Fe–RGO–BiVO_4_ process^36^. The ideal conditions achieved by the CCD approach are shown in Fig. 4c. Under these conditions, prediction of proposed model for the removal efficiency of 2,4-DNT is 100%. Under the identical settings with three replications, the achieved practical removal efficacy of 2,4-DNT was 98.08% ± 0.5. Equation  8 was utilized to calculate the pareto effect of each parameter (Pi) which illustrates each parameter's ability to alter the system response (2,4-DNT elimination efficiency)^30^.

8 $${{\text{P}}}_{{\text{i}}}(\mathrm{\%})=\left(\frac{{\upbeta }_{{\text{i}}}^{2}}{\sum {\upbeta }_{{\text{i}}}^{2}}\right)\times 100$$

where β_i_ represents the regression coefficient for each coded factor in the proposed model's equation. As shown in Fig. 4d, among the suggested parameters, 61.6% of the variations in response of the proposed model are caused by changing 2,4-DNT concentration (22.41%), time (11.79%), pH^2^ (11.63%), Fe–RGO–BiVO_4_ (8.71%) and voltage (7.05%). The most influential independent variable and the most influential interaction effect were belonged to 2,4-DNT and 2,4-DNT * Fe–RGO–BiVO_4_ (AB term), respectively.

### Effect of initial 2,4-DNT concentration

**Figure 5:**
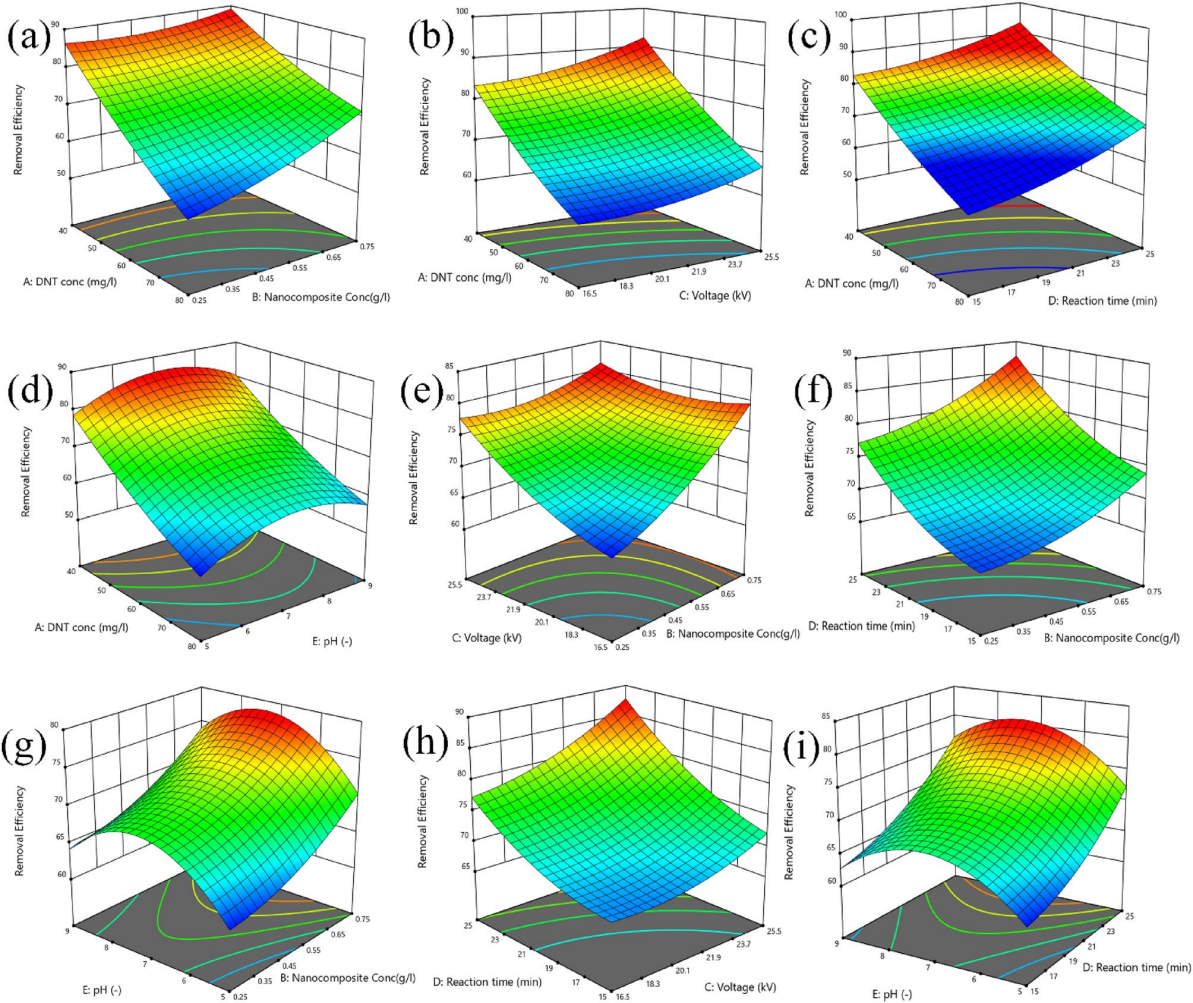
3D response surface plots of the 2.4-DNT removal efficiency, illustrating individual and interactive effects of independent variables on the system response.

**Figure 6:**
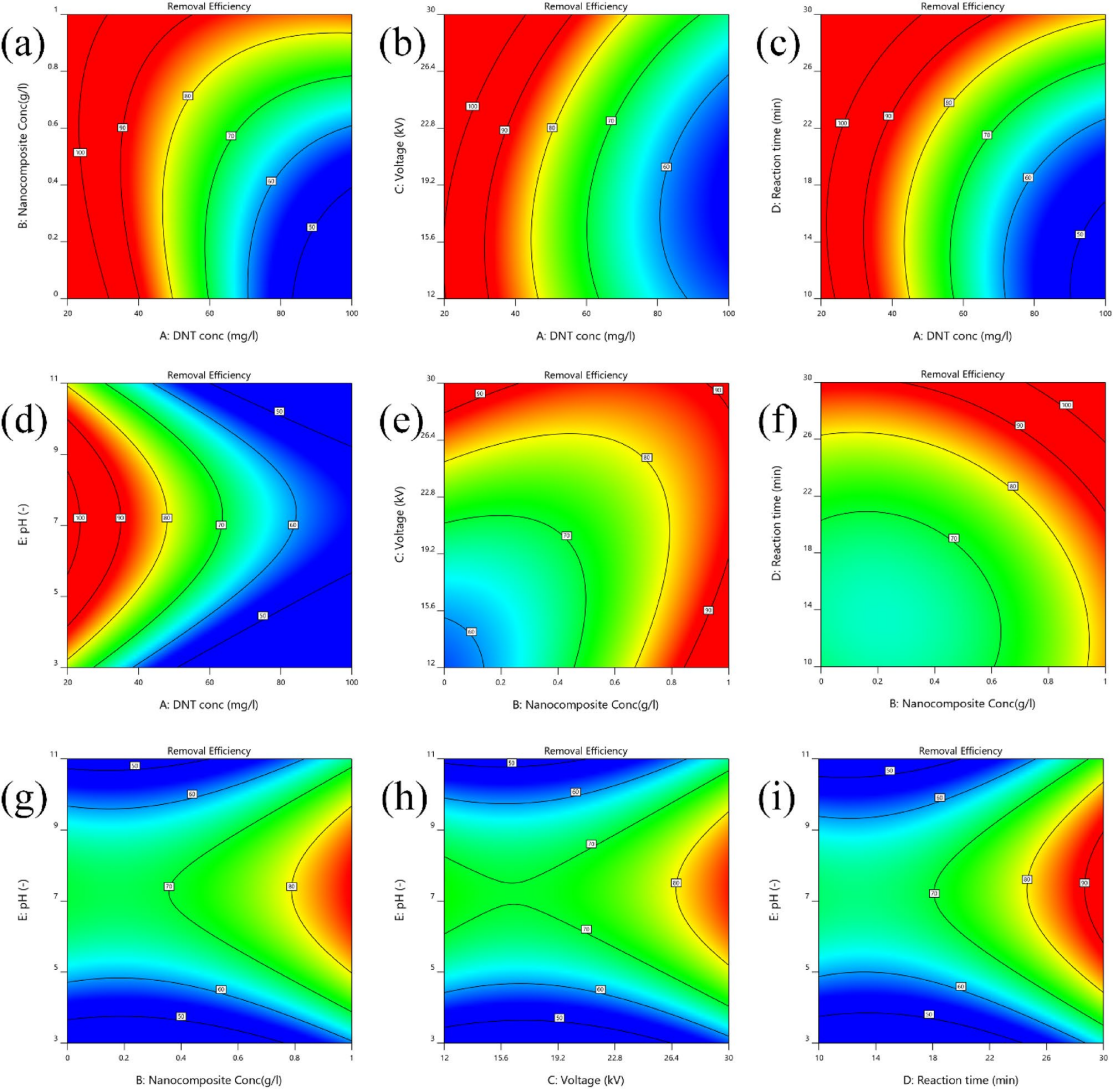
Contour response plots for 2.4-DNT removal rate.

The corresponding pareto effect value for linear term of 2,4-DNT (22.41%) can indicate that this independent variable has the most substantial effect on process efficiency. Also, partial quadratic effect (3.21%) and interaction effect with Fe–RGO–BiVO_4_ (6.22%) were noted for this independent variable. The 3D response surface plots and contour response plots for a Visual representation of the effects of 2,4-DNT concentration regarding to the other parameters are presented in Figs. 5a–d and 6a-d, respectively. It can be stated that the degradation rate was inversely correlated with the initial 2,4-DNT concentration. However, the initial 2,4-DNT concentration lower than 20 mg L^−1^ was not included in the investigation due to design restrictions because of its full removal at lower concentrations. The results represented in Figs. 5a–d and 6a-d indicated that, the interaction of initial 2,4-DNT concentration with other independent variables is in a way that increasing the initial 2,4-DNT concentration from 20 to 100% reduces degradation efficiency from above 90% to less than 60%. However, given that other variables have been maintained at their center point levels (Fe–RGO–BiVO_4_ dosage = 0.5 g L^−1^, Voltage = 21kV, Reaction time = 20 min and pH = 7), when a particular level was reached, the pollutant concentration surpassed the oxidation capacity of DBD/Fe–RGO–BiVO_4_ process which leads to reduced removal efficiency^37,39^. This could be due to the saturation of the catalyst surface by 2,4-DNT molecules, reduction of conductivity and consequently decrease of plasma discharge in the reaction solution, which ultimately reduced the production of reactive species like ${{\text{HO}}}^{\cdot}$ radicals^40^. In fact, the DBD/Fe–RGO–BiVO_4_ process produces a certain number of reactive species that can’t compete with higher concentrations of 2,4-DNT, and so-called reactive species dilution occurs. Also, at higher 2,4-DNT concentrations more intermediate compounds were formed which consumed larger amounts of active species. These results are in good agreement with the findings by Seid-mohammadi et al.^37^ and Wu et al.^35^ regarding the degradation of various organic compounds by AOP processes.

As stated earlier, in the non-thermal plasma reactors the generated electric discharge partially ionizes the inlet gas under the influence of applied high voltage. By applying adequate voltage levels through a dielectric barrier, self-propagation of electrons avalanche, and the generation of reactive species will be achievable. Although, the nature of the feed gas determines the type of produced active species.

In the NTP-DBD reactors, atmospheric air has been frequently employed as the feed gas to produce ozone, one of the most significant oxidizing agents^22^. Ozone production involves a two-step procedure in which electrons (e^-^) generated in the plasma discharge can cause oxygen dissociation and start the first step. In the next step, ozone can be produced via the reaction between dissociated oxygen (O), oxygen (O_2_), and a third collision partner (M), such as other ozone and oxygen molecules, dissociated oxygen (O), and nitrogen (N_2_)^41^. The following is the formation mechanisms of ozone (Eqs. (9–11))^22,41^.

9 $${{\text{e}}}^{-}+{{\text{O}}}_{2}\to {{\text{e}}}^{-}+2{\text{O}}$$

10 $${\text{O}}+{{\text{O}}}_{2}+{\text{M}}\to {{\text{O}}}_{3}+{\text{M}}$$

11 $${{\text{O}}}_{2}+{O}^{\cdot}\to {{\text{O}}}_{3}$$

Equations (12) and (13) presented the ozone reaction mechanisms for hydroxyl radical generation. Also, the reaction of ozone and hydrogen peroxide (${{\text{H}}}_{2}{{\text{O}}}_{2}$) can lead to the production of higher amounts of hydroxyl radicals (${OH}^{\cdot}$), which result in enhanced oxidation of target organic pollutant (Eqs. 14 and 15)^42,43^.

12 $$3{{\text{O}}}_{3}+{{\text{H}}}_{2}{\text{O}}\to 2{{\text{OH}}}^{\cdot}+4{{\text{O}}}_{2}$$

13 $$2{{\text{O}}}_{3}+{{\text{H}}}_{2}{{\text{O}}}_{2}\to 2{{\text{OH}}}^{\cdot}+3{{\text{O}}}_{2}$$

14 $${{\text{O}}}_{3}+{{\text{H}}}_{2}{{\text{O}}}_{2}\to {{\text{OH}}}^{\cdot}+{{\text{HO}}}_{2}^{\cdot}+{{\text{O}}}_{2}$$

15 $${{\text{O}}}_{3}+{{\text{HO}}}_{2}^{\cdot}\to {{\text{O}}}_{2}+{{\text{OH}}}^{\cdot}+{{\text{O}}}_{2}^{-}$$

Moreover, the reaction of ${{\text{O}}}_{3}$ and ${{\text{OH}}}^{\cdot}$ can generate ${{\text{HO}}}_{2}^{\cdot}$, which is a less reactive radical than ${{\text{OH}}}^{\cdot}$ (Eq. 16). However, the produced ${{\text{HO}}}_{2}^{\cdot}$ can produce more ${{\text{OH}}}^{\cdot}$ via reaction with ${{\text{O}}}_{3}$ (Eq. 17)^28,41^.

16 $${{\text{O}}}_{3}+{{\text{OH}}}^{\cdot}\to {{\text{HO}}}_{2}^{\cdot}+{{\text{O}}}_{2}$$

17 $${{\text{O}}}_{3}+{{\text{HO}}}_{2}^{\cdot}\to {{\text{OH}}}^{\cdot}+2{{\text{O}}}_{2}$$

${\text{H}}_{2} {\text{O}}_{2}$ as the other important oxidization agent could be generated by high-energy electrons attacking water molecules in addition to the ${\text{O}}_{3}$ oxidation of water molecules. Moreover, ${\text{H}}_{2} {\text{O}}_{2}$ can be generated by free radicals^44^. The following express the ${\text{H}}_{2} {\text{O}}_{2}$ reaction formula (Eqs. 18–23).

18 $${\text{e}}^{ - } + {\text{H}}_{2} {\text{O}} \to {\text{OH}}^{ \cdot } + {\text{H}}^{ \cdot } + {\text{e}}^{ - }$$

19 $${\text{OH}}^{ \cdot } + {\text{OH}}^{ \cdot } \to {\text{H}}_{2} {\text{O}}_{2}$$

20 $$2{\text{H}}_{2} {\text{O}} \to {\text{H}}_{2} + {\text{H}}_{2} {\text{O}}_{2}$$

21 $${\text{O}}_{2} + {\text{H}}^{ \cdot } \to {\text{HO}}_{2}^{ \cdot }$$

22 $${\text{HO}}_{2}^{ \cdot } + {\text{H}}^{ \cdot } \to {\text{H}}_{2} {\text{O}}_{2}$$

23 $${\text{HO}}_{2}^{ \cdot } + {\text{HO}}_{2}^{ \cdot } \to {\text{O}}_{2} + {\text{H}}_{2} {\text{O}}_{2}$$

Moreover, it has also been reported that ${\text{H}}_{2} {\text{O}}_{2}$ can be re-decomposed into ${\text{OH}}^{ \cdot }$ under the UV radiations generated by the plasma, which is conducive to the effective degradation of 2,4-DNT (Eqs. (24) and (25))^44,45^.

24 $${\text{H}}_{2} {\text{O}}_{2} + h\nu \to {\text{OH}}^{ \cdot } + {\text{OH}}^{ \cdot }$$

25 $${\text{H}}_{2} {\text{O}} + h\nu \to {\text{OH}}^{ \cdot } + {\text{H}}^{ \cdot }$$

### Effect of Fe–RGO–BiVO_4_ nanocomposite

**Figure 7:**
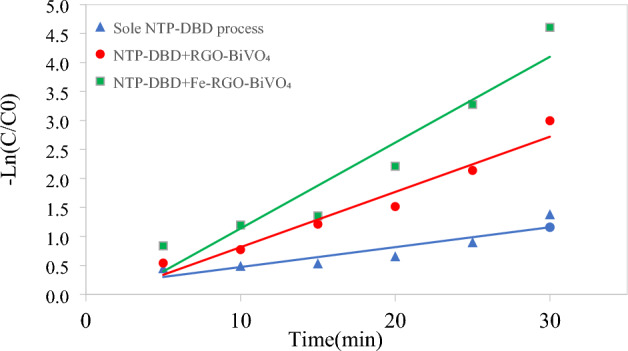
The kinetic plots (pseudo-first order) of the sole NTP-DBD, DBD/RGO-BiVO_4_ and DBD/Fe–RGO–BiVO_4_ processes (initial 2.4-DNT concentration = 80 mg/L, Fe–RGO–BiVO_4_ dosage = 0.5 g/L, Voltage = 21kV, Reaction time = 30 min and pH = 7).

Figures 5a,e,f, and g and 6a,e,f, and g show the interaction of the Fe–RGO–BiVO_4_ dosage and other independent variables on 2,4-DNT removal efficiency in the DBD/Fe–RGO–BiVO_4_ system. As can be seen in Figs. 5e and 6e, at initial 2,4-DNT concentration of 60 mg L^−1^, reaction time of 20 min and pH = 7, when Fe–RGO–BiVO_4_ concentration and applied voltage increased the removal efficiency of 2,4-DNT gradually increased. This may be due to a high concentration of reactive species (e.g., ${\text{H}}_{2} {\text{O}}_{2}$, ${\text{O}}_{3}$, etc.) and production of the ${\text{OH}}^{\cdot }$ radicals because of enhanced plasma discharge and photocatalyst activation. On the other hand, increasing each of these two independent variables reduces the effect of the other variable on the removal efficiency. At a photocatalyst concentration of 0.25 mg L^−1^, increasing the applied voltage from 12 to 30 kV increased the removal efficiency by 17.5%, but at a photocatalyst concentration of 0.75 mg L^−1^, it only increased by 2.5%. This may be because the highest process efficiency (85%) occurs at 0.75 mg L^−1^ of Fe–RGO–BiVO_4_ concentration and a further increase due to voltage change is not possible. These findings were supported by other investigations^25,35^. The greater photocatalyst concentrations, according to the research of Seid-Mohammadi et al., which provide more absorption and reaction surface as well as more concentrations of iron species to form ${\text{HO}}^{\cdot }$ radicals^37^. To ascertain the rate of 2,4-DNT adsorption by synthesized photocatalyst, dark environment adsorption tests were conducted. The 2,4-DNT adsorption rate by 1 g L^−1^ of Fe–RGO–BiVO_4_ after 60 min of contact time were less than 7 ± 1.8% under optimal conditions. Considering that the highest reaction time selected for the current research was 30 min, the effects of adsorption could be ignored. In order to evaluate the effect of Fe–RGO–BiVO_4_ composite on NTP-DBD process's efficiency, a kinetic analysis conducted. The pseudo-first order kinetic plot of the processes under optimized model conditions (initial 2,4-DNT concentration = 80 mg L^−1^, Fe–RGO–BiVO_4_ dosage = 0.75 g L^−1^, Voltage = 21kV, Reaction time = 30 min and pH = 7) is shown in Fig. 7. Also, the pseudo-first order kinetic constants (K_obs_), determined by calculating the slope of the drawn line of Ln (C/Co) versus time^44^, are shown in Table 4. Whereas the K_obs_ of the sole NTP-DBD reaction was 0.034 min^−1^, adding Fe–RGO–BiVO_4_ (0.75 g L^−1^) increased oxidation rate to 0.148 min^−1^. Kinetic study findings from sole NTP-DBD process are in line with other studies^37–42^.

**Table 4:** Degradation kinetics (pseudo first-order model) constants of 2,4-DNT removal by sole NTP-DBD, DBD/RGO-BiVO_4_ and DBD/Fe–RGO–BiVO_4_ processes (initial 2,4-DNT concentration = 80 mg/L, Fe–RGO–BiVO_4_ dosage = 0.5 g/L, Voltage = 21kV, Reaction time = 30 min and pH = 7).

Kinetic parameters	K_obs_ (min^−1^)	R-square
Sole NTP-DBD process	0.0343	0.8137
NTP-DBD + RGO-BiVO_4_	0.0953	0.9521
NTP-DBD + Fe-RGO-BiVO_4_	0.1481	0.9141

Also, it is documented in the literature that adding 0.75 g L^−1^ of Fe–RGO–BiVO_4_ nanocomposite increased the rate of oxidation. In a study by Ahmadi et al. (2021), the introduction of 0.5 g L^−1^ α-Fe_2_O_3_-TiO_2_ composite to an NTP-DBD reactor enhanced the pseudo-first order kinetic constant of DMP deterioration by 0.041 min^−1^^45^. Furthermore, 0.75 g L^−1^ of Fe–RGO–BiVO_4_ nanocomposite exhibits remarkable photocatalytic activity. Application of 0.75 g L^−1^ of RGO-BiVO_4_ and 0.75 g L^−1^ of Fe–RGO–BiVO_4_ led to K_obs_ values of 0.095 and 0.148 min^−1^, respectively. Hence, it could be deduced that the addition of Fe and RGO improved the performance of the BiVO_4_ photocatalyst.

**Figure 8:**
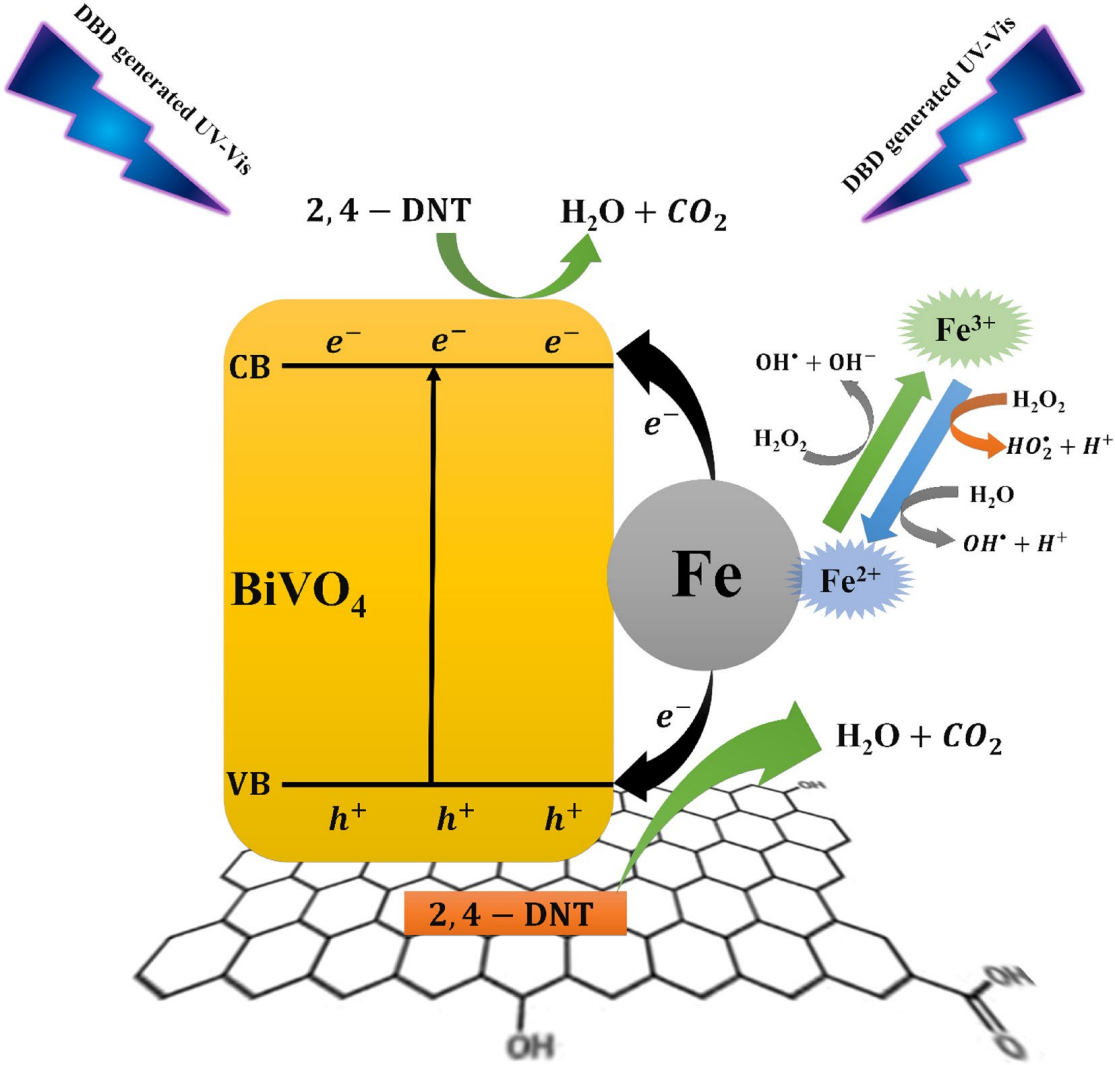
The proposed photocatalytic activity of Fe–RGO–BiVO_4_ and electronic transition between Fe and RGO-BiVO4.

According to the observed properties of synthesized Fe–RGO–BiVO_4_ nanocomposite (mainly XRD and DRS), the feasible photocatalytic activity could be suggested as shown in Fig. 8. The typical photocatalytic activity of BiVO_4_ is represented by Eq. (26) in which, electrons ($e_{{{\text{cb}}}}^{ - }$) of the conduction band (CB) and holes ($h_{{{\text{vb}}}}^{ + }$) of the valance band (VB) may be produced by photo-excitation of the metal oxide semiconductor^25^. The adsorbed water (Eq. (27)) and hydroxyl ions (-OH) (Eq. (28)) can be directly oxidized by valence band holes of the catalyst to generate the hydroxyl radical (${\text{HO}}^{ \cdot }$).

26 $${\text{BiVO}}_{4} + h\nu \to {\text{BiVO}}_{4} \left( {e_{cb}^{ - } + h_{vb}^{ + } } \right)$$

27 $${\text{BiVO}}_{4} \left( {h_{vb}^{ + } } \right) + H_{2} O \to {\text{BiVO}}_{4} + OH^{ \cdot } + H^{ + }$$

28 $${\text{BiVO}}_{4} \left( {h_{vb}^{ + } } \right) + OH^{ - } \to {\text{BiVO}}_{4} + OH^{ \cdot }$$

Also, the generated photoinduced electrons in the CB of the catalyst can reduce molecular oxygen to hydroperoxyl radical (${\text{HO}}_{2}^{^\circ }$) that is the protonated form of superoxide (Eq. (29,30)). Moreover, presence of Fe^3+^ ions causes effective charge separation and makes it easier to produce ${\text{O}}_{2}^{ \cdot - }$ from oxygen molecules^45^. The generated ${\text{O}}_{2}^{ \cdot - }$, will lead to ${\text{H}}_{2} {\text{O}}_{2}$ and ${\text{OH}}^{ \cdot}$ production (Eq. 31–33)^31,32^

29 $$e_{cb}^{ - } + {\text{O}}_{2} \to {\text{O}}_{2}^{ \cdot - }$$

30 $${\text{O}}_{2}^{ \cdot - } + {\text{H}}^{ + } \to {\text{HO}}_{2}^{ \cdot }$$

31 $$2{\text{HO}}_{2}^{\cdot } \to {\text{H}}_{2} {\text{O}}_{2} + {\text{O}}_{2}$$

32 $${\text{H}}_{2} {\text{O}}_{2} + {\text{O}}_{2}^{ \cdot - } \to {\text{OH}}^{ \cdot } + {\text{OH}}^{ - } + {\text{O}}_{2}$$

33 $$e_{cb}^{ - } + {\text{H}}_{2} {\text{O}}_{2} \to {\text{OH}}^{ \cdot } + {\text{OH}}^{ - }$$

It is clearly seen that RGO-BiVO_4_ and Fe-loaded RGO-BiVO_4_ exhibited higher photoactivity than pure BiVO_4_ in NTP-DBD process. This is possibly due to the enhanced light absorption intensity of the Fe–RGO–BiVO_4_ as emphasized by UV–vis diffuse reflectance spectra in Fig. 2b. The increased photocatalytic activity could also be attributed to Fe^3+^ ions acting as both electron and hole traps, that increases the lifetimes of electrons and holes and reduces the e^−^/h^+^ pair recombination rate^45–47^. The adsorbed target pollutant can be degraded through direct oxidation by $h_{vb}^{ + }$ on the catalyst surface or by ${\text{OH}}^{ \cdot }$ generation (Eqs. (34, 35))^45^.

34 $$h_{vb}^{ + } + {\text{organic pollutant}} \to {\text{organic pollutant}}^{ \cdot - } \to {\text{oxidized products}}$$

35 $$OH^{ \cdot } + {\text{organic pollutant}} \to {\text{oxidized products}}$$

Many literature reviews^37,47^ have also proposed that in addition to producing ${\text{OH}}^{ \cdot }$ through heterogeneous pathways, metal cation (Fe^3+^) can also produce ${\text{OH}}^{ \cdot }$ through an alternative Fenton-type reaction (Eq. (36)).

36 $$Fe^{{\left( {n - 1} \right) + }} + H_{2} O_{2} + H^{ + } \to Fe^{n + } + OH^{ - } OH^{ \cdot }$$

Likewise, Other reactive species produced by plasma discharge, including electrons and $H_{2} O_{2}$ can replenish iron's active state (Fe^2+^) in catalytic reactions and Fenton-type process by the Fe^3+^ reduction reactions (Eqs. 37–39)^37,48^. The electrons produced in NTP reactor are very energetic (1–10 eV)^45,48^. This provides a rational justification for understanding the cause of Fe's effectiveness at the nanocomposite structure in the NTP-DBD process.

37 $${\text{Fe}}^{3 + } + {\text{H}}_{2} {\text{O}}_{2} \to {\text{Fe}}^{2 + } + {\text{H}}^{ + } + {\text{HO}}_{2}^{ \cdot }$$

38 $${\text{Fe}}^{3 + } + {\text{HO}}_{2}^{ \cdot} \to {\text{Fe}}^{2 + } + {\text{H}}^{ + } + {\text{O}}_{2}$$

39 $${\text{Fe}}^{3 + } + {\text{ e}}^{ - } \to {\text{Fe}}^{2 + }$$

Every one of these pathways may improve photocatalytic activity of Fe–RGO–BiVO_4_ in NTP-DBD process. Besides, due to its wide surface area and capacity for electron storage, RGO can potentially absorb more 2,4-DNT, and the resulting electron accumulation could favorably react with 2,4-DNT as the target electron acceptor. Also, doped Fe acted as an electron donor, interacting with an electron mediator to generate a large number of electrons while also making the charge transfer process easier. The RGO served as an efficient platform for 2,4-DNT absorption while also supplying adequate electrons for 2,4-DNT reduction. As a result, in the presence of Fe and RGO, co-modified BiVO_4_ could obtain effective generation, transportation and mobility of electrons, leading to enhanced 2,4-DNT degradation.

### Effect of the applied voltage

The influence of applied voltage and any potential interactions between it and the other chosen independent variables were evaluated in the 12 to 30 kV range. The 2,4-DNT degradation efficiency was positively impacted by applied voltage in a linear way with pareto effect of 7.05% and F-value of 248.28. This indicates that the overall efficacy could be greatly affected by the applied voltage. The response surface and contour response plots of applied voltage changes under various operating circumstances are displayed in Figs. 5b, e, and h and 6b, e, and h. It should be noted that even though the plasma arc formation was seen at the lowest selected discharge voltage (12 kV), it was discovered that only around 67.1% of the 2,4-DNT could be eliminated at this applied voltage and at the optimal points (initial 2,4-DNT concentration = 60 mg L^−1^, Fe–RGO–BiVO_4_ dosage = 0.5 g L^−1^, Reaction time = 20 min and pH = 7). As shown in Figs. 5h and 6h, under identical operating conditions, raising the discharge voltage from 12 to 30 kV got a significant impact on removal efficiency of 2,4-DNT, leading to a 78% removal efficiency. The similar finding was observed in a prior investigation by Chen et al. (2009), whereby a plate-shaped DBD reactor's efficiency was sharply enhanced by raising the applied voltage from 2.5 to 3 kV^49^. This improvement can be ascribed to the increased rate of electron production and the abundancy of active species, that may raise the impact and reaction possibility of 2,4-DNT with active species^46^. The significant correlation between the discharge voltage and the concentration of Fe–RGO–BiVO_4_ with pareto effect of 5.56% and F-value of 123.53 can illustrate that the applied voltage influences the photocatalyst excitation in a favorable way. This could be due to increased UV radiation (Eq. 7), which has led to increased generation of active species such as electrons and $H_{2} O_{2}$. Another interaction effect discovered for discharge voltage was its correlation with reaction time. As shown in Fig. 5h, increasing the applied voltage reduces the reaction time required for removing 2,4-DNT. Moreover, at higher applied voltages, more ${\text{Fe}}^{2 + }$ can be regenerated due to increased UV radiation and increased generation of active species such as $H_{2} O_{2}$ and O_3_. Therefore, more reactive species such as $OH^{ \cdot }$ are produced in the catalytic process of.

### Effect of reaction time

The pareto effect of 11.79% and F-value of 694.42 for the reaction time indicated that this independent variable is the second most important factor in for determining the of Fe–RGO–BiVO_4_ process efficiency. However, for this independent variable, no interaction or quadratic effects were found. Figures 5c, f, h and i and 6c, f and i provide response surface and contour response plots to illustrate the interaction of reaction time with other parameters. These figures make it clear that the reaction time had a positive impact on the process efficiency because the efficacy considerably rose as the reaction time was increased. Along with this, there is a clear positive interaction between reaction time and the other independent factors. As it can be seen in Figs. 5g and 6g, when the initial catalyst dosage, 2,4-DNT concentration, pH and applied voltage respectively were 0.5 g L^−1^, 60 mg L^−1^, 7, and 25 kV, by raising the reaction time from 10 to 30 min the removal efficiency of 2,4-DNT increased from 65 to 77%. Moreover, under optimized model conditions (initial 2,4-DNT concentration = 80 mg L^−1^, Fe–RGO–BiVO_4_ dosage = 0.75 g L^−1^, Voltage = 21kV, and pH = 7) complete removal of 2,4-DNT attained after 30 min. The effect of reaction time can be due to the increased generation of active species and increased exposure of 2,4-DNT to reactive species. Regarding the studied range of reaction time, the current study's findings are promising as significant efficacy was attained in less than 15 min of reaction time. As mentioned before, in comparison to earlier investigations, the current reaction time findings are promising. For instance, to completely degrade 22.5 mg L^−1^ of methyl violet by DBD plasma at 30 kV of discharged voltage, 30 min of reaction time were needed. 30 min of reaction time at 30 kV of applied voltage for the complete degradation of 22.5 mg L^−1^ of methyl violet by DBD plasma required^47^. Similarly, according to Magureanu et al. (2009), it took 30 min to remove 95% of methylene blue (50 mg L^−1^) with 12 kV of discharge voltage in the DBD plasma reactor with falling film flow pattern. Magureanu et al. (2009) reported that in the falling film DBD plasma reactor, it took 30 min to remove 95% of 50 mg L^−1^ of methylene blue with 12 kV of discharge voltage^50^.

### Effect of pH

The pH level of reaction media is a key variable in organic pollutant degradation as it may have an impact on the target pollutant's ionization potential, the production of charged radicals, the conduction band potential and the photocatalyst's surface characteristics^51^. ${\text{H}}_{2} {\text{O}}_{2} ,{\text{ O}}_{3} ,{\text{and OH}}^{ \cdot }$ are the most dominant active species generated in plasma reactors. The pH level of the reaction media can have an effect on the formation (type, role, and quantity) of mentioned active species. The formation of these active species, may be impacted by the by pH level of reaction media^52^. According to Fig. 4d, pH value with pareto effect of 11.63% and F-value of 432.48 showed a quadratic effect on the model response. The interactions of pH with other factors are shown in Fig. 5d, g and i and 6d, g, h and i. Accordingly, at central points of independent variables (2,4-DNT concentration = 60 mg L^−1^, Fe–RGO–BiVO_4_ dosage = 0.5 g L^−1^, voltage = 21kV, and time = 20 min) when sample pH was raised from 3 to 11, the degradation efficiency initially increased and subsequently declined, while the maximum efficiency (80.5%) was achieved at the pH 7. This implies that in comparison to acidic or alkaline environments, neutral pH (= 7) provides more suitable conditions to attain better decomposition efficiency. It's also crucial to note that the negative effect of alkaline pH on process efficiency was more than acidic pH. The results of some earlier studies have also confirmed that greater amount of ${\text{H}}^{ + }$‏or ${\text{OH}}^{ - }$ in the reaction media would have an adverse effect on the photocatalytic process^32,53^. Mahlalela et al. have stated that the reason for this could be connected to the surface charge of the photocatalyst and the pollutant^54^. Interplay between the pollutant and the nanocomposites would occur at pH levels where the pollutant had a negative surface charge while the nanocomposites were positively charged, or vice versa. In acidic conditions, the functional groups were protonated and 2,4-DNT surface were positively charged due to protonation. When the pH moves closer to neutral, 2,4-DNT's protonation declines and that enfeebles the electrical repulsive forces between the positively charged nanocomposites. The reduction of process efficiency at alkaline environments (relative to the acidic conditions) could be due to the detrimental effect of hydroxyl anion (${\text{OH}}^{ - }$) on the strong reaction between ${\text{ OH}}^{ \cdot }$ and 2,4-DNT^55^. Furthermore, decomposition of ${\text{H}}_{2} {\text{O}}_{2}$ under high pH levels generates hydro peroxide anion (${\text{HO}}_{2}^{ - }$) which has the ability to act as a ${\text{ OH}}^{ \cdot }$ quenching agent^25^. Besides, at acidic solutions, the reactions induced by ${\text{HO}}_{2}^{ \cdot }$, ${\text{O}}_{2}^{ \cdot - }$ and generated electrons during plasma discharge, higher amounts of hydroxyl radicals can be generated^55^. It must be highlighted that the achieved performance under alkaline and acidic conditions is yet desirable, and the NTP-DBD technique could thus be applicable for a wide variety of pH levels. Based on the literature, it can be stated that higher process efficiency at pH = 7 might have caused by the accumulation of excess hydroxyl radicals (${\text{OH}}^{ \cdot }$) on the photocatalyst surface^56–58^. Even though the capacity of ${\text{O}}_{3}$ to degrade contaminants can be hampered in acidic conditions, there have been reports that the ${\text{O}}_{3}$ molecule could easily combine with ${\text{OH}}^{ - }$ in alkaline solutions to generate ${\text{OH}}^{ \cdot }$, which can degrade the contaminant under alkaline conditions. In a prior investigation, the same finding was also obtained^45^.

### Biodegradability and mineralization

In terms of improving biodegradability, the DBD/Fe–RGO–BiVO_4_ system had shown favorable outcome. Moreover, the average oxidation state (AOS) and carbon oxidation state (COS) have been determined using Eqs. (40) and (41), respectively, to assess the level of oxidation and efficacy of the oxidative process. As may be seen, to calculate those indicators, the COD and DOC values have been measured at several reaction times^59,60^.

40 $${\text{Average Oxidation State}} = \left( 4 \right) - \left( {1.5\frac{COD}{{DOC}}} \right)$$

41 $${\text{Carbon Oxidation State}} = \left( 4 \right) - \left( {1.5\frac{COD}{{DOC_{0} }}} \right)$$

**Figure 9:**
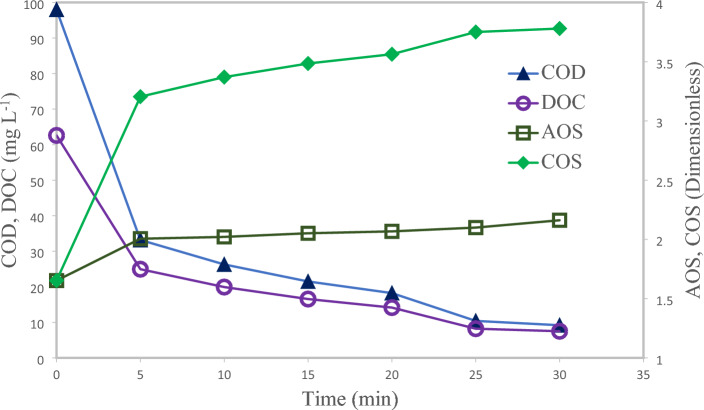
Changes of AOS, COS, COD and DOC in relation to reaction time during the DBD/Fe–RGO–BiVO_4_ process. (Initial concentration of 2.4-DNT = 80 mg/L, Fe–RGO–BiVO_4_ dosage = 0.75 g/L, Voltage = 21 kV, and pH = 7).

DOC stands for dissolved organic carbon at sampling time of *t* (mgC L^−1^), DOC_0_ stands for dissolved organic carbon of the solution at sampling time of 0 (mgC L^−1^), and COD stands for chemical oxygen demand at sampling time of *t* (mgO_2_ L^−1^). The AOS value varies between + 4 and − 4 with the highest value (+ 4) representing CO_2_, the most oxidized form of Carbon, and the lowest value (− 4) representing CH_4_, the most reduced form of Carbon. Only the solution's organic compounds are taken into account by the AOS^59,60^^.^ It's important to point out that in COS calculations, even the $CO_{2}$ that is removed from the reaction solution is included^60–62^. Accordingly, since AOS and COS are indicative of qualitative changes in the solution that result in toxicity and biodegradability, they can serve as indirect indicators of biodegradability. Figure 9 illustrates the variations in AOS, COS, DOC, and COD values that occurred during the DBD/Fe–RGO–BiVO_4_ process. Process efficiency in 2,4-DNT mineralization can be depicted by the DOC variation curve. In the case of DOC, it is evident that as the reaction time passed, the concentration reduced until after 30 min, 88.02% of the DOC was removed. The high level of DOC removal indicates that 2,4-DNT had been substantially mineralized and deteriorated, and the final solution contains negligible amounts of intermediates. Additionally, a rise in the AOS level from 1.65 to 2.16 and the COS level from 1.65 to 3.78 show that the 2,4-DNT has been heavily mineralized, and the biodegradability of the solution had been enhanced. The significant oxidation of 2,4-DNT is shown by a 90.62% drop in COD content. As well, an increase in the AOS value from 1.65 to 2.16 and the COS value from 1.65 to 3.78 demonstrate that the 2,4-DNT has been significantly mineralized, and the solution biodegradability has been enhanced. Strong oxidation of 2,4-DNT is indicated by a 90.62% drop in COD content.. This was further confirmed by variations in the COS values over the course of the process, where the starting value of 1.65 shows the presence of 2,4-DNT as a reduced organic molecule, and the ultimate value of 3.78 confirms the significant mineralization and formation of highly oxidized intermediate products. It can be obtained from AOS curve which AOS value of 1.65 indicates that the process starts with more reduced molecules, and its growth to 2.00 after 5 min indicates the production of oxidized intermediates. An increase in the AOS with a gentle slope from the 5th to the 30th minute indicates a slight increase in oxidized species versus reduced species. After 10 min, the AOS value had reached a plateau between 2.00 and 2.16, which indicated that the chemicals and intermediates have not undergone any substantial change during this time^63^. Several oxidation-mineralization pathways can be considered for the degradation of 2,4-DNT. By considering all the aspects, based on the observed intermediates and their changes, it may be concluded that 2,4-DNT degradation and mineralization occurred under a variety of oxidation-mineralization pathways producing highly mineralized products.

### Proposed degradation pathway

**Figure 10:**
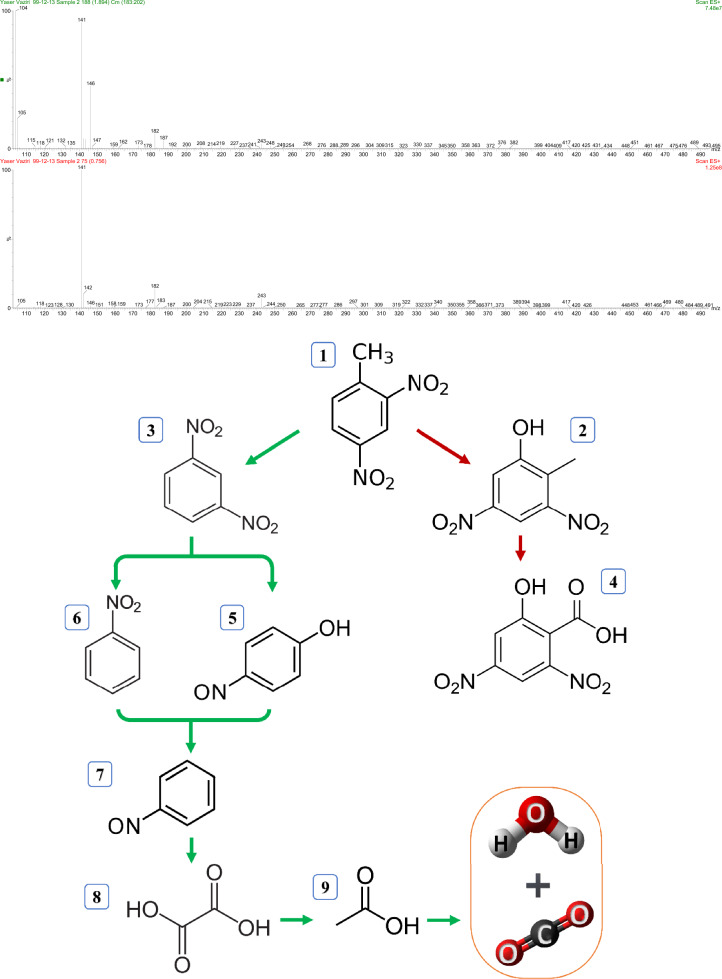
LC–MS chromatographs and suggested 2,4-DNT degradation pathway by DBD/Fe–RGO–BiVO_4_ under optimal conditions.

For determination of intermediates produced throughout the decomposition process and their degradation mechanisms, the DBD/Fe–RGO–BiVO_4_ system was conducted at optimum settings (initial 2,4-DNT concentration = 40 mg L^−1^, Fe–RGO–BiVO_4_ dosage = 0.75 g L^−1^, Voltage = 21kV, Reaction time = 30 min and pH = 7). Through following up the DBD/Fe–RGO–BiVO_4_ reactor by a liquid chromatography-mass spectrometry (LC–MS), degradation intermediates have been recognized. The data shown in Fig. 10. It was found that masses over 182 were associated with the compound's binding to hydroxide and the formation of 2-methyl-3,5-dinitrophenol. Furthermore, the methyl functional group was oxidized to produce 2-hydroxy-4,6-dinitrobenzoic acid, which has a mass of 228. The major reaction involves isolating a methyl group, followed by forming 1, 3-dinitrobenzene with a mass of 168. When the nitro group is removed, compounds, such as nitrobenzene or 4-nitrosophenol, are formed. Next, the nitrosobenzene molecule with a mass of 107 was changed to produce acetic acid, followed by the production of oxalic acid (with a mass of 60). Eventually all these compounds with lower molecular masses are converted to carbon dioxide and water.

## Conclusions

In the present work, newfound visible-light photocatalyst of Fe–RGO–BiVO_4_ was synthesized and evaluated as a potential promoting agent to improve the NTP-DBD reactor's capability for efficient 2,4-DNT degradation. The results of XRD, DRS, EDX mapping, and FESEM affirmed the satisfactory deposition of Fe and RGO on the BiVO_4_ surface. The catalytic plasma process exhibited an improved efficiency and Fe–RGO–BiVO_4_ nanocomposite had a synergistic influence on the process's efficiency. A quadratic model with an R^2^ value higher than 0.99 was obtained based on RSM-CCD to forecast the degradation efficiency of 2,4-DNT. According to this model, the optimal process conditions for initial 2,4-DNT concentration, Fe–RGO–BiVO_4_ dosage, applied voltage, Reaction time and pH were 40 mg L^−1^, 0.75 g L^−1^, 21 kV, 30 min, and 7, respectively. By applying these settings, the TOC, COD, and 2,4-DNT removal efficiencies in catalytic plasma reactor were 88.02%, 90.62%, and 99.5%, respectively. The results of AOS and COS showed that DBD/Fe–RGO–BiVO_4_ process increased the biodegradability of the effluent. Considering the findings, the catalytic plasma process can reduce the reaction time significantly. However, in order for the process to be successful, certain conditions need to be met. Despite the fact that each of the selected variables had a statistically significant effect on the process efficiency, direct effect of discharged voltage and its interactions with Fe–RGO–BiVO_4_ concentration had an important role in the efficient removal of 2,4-DNT. The results indicated that the studied pollutant (2,4-DNT) was entirely reduced to CO_2_ and H_2_O by the catalytic plasma process, and no hazardous or detrimental byproducts were found. The present study can provide new insights into the use of the catalytic plasma process for remediation purposes in aqueous media.

## Data Availability

All experimental data were published in the current article. To additional data and information will be provided to individuals upon official request to the corresponding authors [Abdolmotaleb Seidmohammadi].

## References

[1] Chen WS, Hsu MC (2023). Ultrasound-assisted mineralization of 2,4-dinitrotoluene in industrial wastewater using persulfate coupled with semiconductors. Molecules..

[2] Su L (2023). 2,4-Dinitrotoluene (2,4-DNT) exposure induces liver developmental damage and perturbs lipid metabolism and oxygen transport gene expression in zebrafish (*Danio rerio*). Environ. Sci. Poll. Res..

[3] USEPA. Priority pollutant list. United State Environmental Protection Agency. (2014). Available in: https://www.epa.gov/sites/default/files/2015-09/documents/priority-pollutant-list-epa.pdf

[4] Ishaque AB (2005). Cytotoxicity of dinitrotoluenes (2, 4-DNT, 2, 6-DNT) to MCF-7 and MRC-5 cells. Int. J. Environ. Res. Public Health..

[5] Gumuscu B, Tekiany T (2023). Effective biodegradation of 2,4,6-trinitrotoluene using a novel bacterial strain isolated from TNT-contaminated soil. Int. Biodeter. Biodeger..

[6] Paden NE, Smith EE, Kendall RJ (2008). Acute toxicity of 2, 4, 6-trinitrotoluene, 2, 4-dinitrotoluene, and 2, 6-dinitrotoluene in the adult bullfrog (Lithobates catesbeiana). Bull. Environ. Contam. Toxicol..

[7] Debasree K, Chinmay H, Ambalal C (2015). Isolation, screening and assessment of microbial isolates for biodegradation of 2, 4-and 2, 6-dinitrotoluene. Int. J. Curr. Microbiol. App. Sci..

[8] Wang X, Qian P, Song K, Zhang C, Dong J (2023). The DFT study of adsorption of 2,4-dinitrotoluene on kaolinite surfaces. Comput. Theor. Chem..

[9] Monti MR, Smania AM, Fabro G, Alvarez ME, Argarana CE (2005). Engineering Pseudomonas fluorescens for biodegradation of 2, 4-dinitrotoluene. Appl. Environ. Microbiol..

[10] Guo X (2021). Effective visible-light excited charge separation in all-solid-state Ag bridged BiVO_4_/ZnIn_2_S_4_ core-shell structure Z-scheme nanocomposites for boosting photocatalytic organics degradation. J. Alloys. Compd..

[11] Ghorbanian Z, Asgari G, Samadi MT, Seid-Mohammadi A (2019). Removal of 2,4 dichlorophenol using microwave assisted nanoscale zero-valent copper activated persulfate from aqueous solutions: Mineralization, kinetics, and degradation pathways. J. Mol. Liq..

[12] Samsudin MF, Mahmood A, Sufian S (2018). Enhanced photocatalytic degradation of wastewater over RGO-TiO_2_/BiVO_4_ photocatalyst under solar light irradiation. J. Mol. Liq..

[13] Wang X, Hu S, Mao H, Wei X, Naraginti S (2023). Facile fabrication of AgVO_3_/rGO/BiVO_4_ hetero junction for efficient degradation and detoxification of norfloxacin. Environ. Res..

[14] Selvakumar K (2023). Fabrication of single tungsten / copper / cobalt atom oxide anchored BiVO_4_-rGO for boosting photodegradation of norfloxacin and rhodamine B. J. Clean. Prod..

[15] Lakhera SK, Hafeez HY, Venkataramana R, Veluswamy P, Choi H, Neppolian B (2019). Design of a highly efficient ternary AgI/rGO/BiVO_4_ nanocomposite and its direct solar light induced photocatalytic activity. Appl. Sur. Sci..

[16] Samy M, Mensah K, El-Fakharany EM, Elkady M, Shokry H (2023). Green valorization of end-of-life toner powder to iron oxide-nanographene nanohybrid as a recyclable persulfate activator for degrading emerging micropollutants. Environ Res..

[17] Mensah K, Mahmoud H, Fujii M, Samy M, Shokry H (2022). Dye removal using novel adsorbents synthesized from plastic waste and eggshell: mechanism, isotherms, kinetics, thermodynamics, regeneration, and water matrices. Biomass Convers. Biorefin..

[18] Regmi C, Kshetri YK, Kim TH, Dhakal D, Lee SW (2019). Mechanistic understanding of enhanced photocatalytic activity of N-doped BiVO_4_ towards degradation of ibuprofen: an experimental and theoretical approach. Mol. Catal..

[19] Nguyen TD (2020). BiVO_4_ photocatalysis design and applications to oxygen production and degradation of organic compounds: a review. Environ. Chem. Let..

[20] Seid-Mohammadi A, Asgari G, Rafiee M, Samadi MT, Nouri F, Pirsaheb M (2022). Fate and inhibition of Bis (2-Ethylhexyl) phthalate in biophysical reactors for treating real landfill leachate. Process. Saf. Environ. Prot..

[21] Magureanu M, Mandache NB, Parvulescu VI (2015). Degradation of pharmaceutical compounds in water by non-thermal plasma treatment. Water. Res..

[22] Aziz KH (2019). Removal of dichloroacetic acid from aqueous solution using non-thermal plasma generated by dielectric barrier discharge and nano-pulse corona discharge. Sep. Purif. Technol..

[23] Wu H, Fan J, Chen W, Yang C (2020). Dielectric barrier discharge-coupled Fe-based zeolite to remove ammonia nitrogen and phenol pollutants from water. Sep. Purif. Technol..

[24] Jahani F, Maleki B, Mansouri M, Noorimotlagh Z, Mirzaee SA (2023). Enhanced photocatalytic performance of milkvetch-derived biochar via ZnO–Ce nanoparticle decoration for reactive blue 19 dye removal. Sci. Rep..

[25] Dehdar A, Asgari G, Leili M, Madrakian T, Seid-Mohammadi A (2021). Step-scheme BiVO_4_/WO_3_ heterojunction photocatalyst under visible LED light irradiation removing 4-chlorophenol in aqueous solutions. J. Environ. Manage..

[26] Hummers WS, Offeman RE (1958). Preparation of graphitic oxide. J. Am. Chem. Soc..

[27] Tian N (2023). Peroxymonosulfate assisted pesticide breakdown: Unveiling the potential of a novel S-scheme ZnO@CoFe_2_O_4_ photo-catalyst, anchored on activated carbon. Environ. Pollut..

[28] Isari AA (2020). Sono-photocatalytic degradation of tetracycline and pharmaceutical wastewater using WO_3_/CNT heterojunction nanocomposite under US and visible light irradiations: A novel hybrid system. J. Hazard. Mater..

[29] Ocholi O, Menkiti M, Auta M, Ezemagu I (2018). Optimization of the operating parameters for the extractive synthesis of biolubricant from sesame seed oil via response surface methodology. Egypt. J. Pet..

[30] Bezerra MA, Santelli RE, Oliveira EP, VillarEscaleira LA (2008). Response surface methodology (RSM) as a tool for optimization in analytical chemistry. Talanta..

[31] Li H (2015). Hierarchically Z-scheme photocatalyst of Ag@ AgCl decorated on BiVO_4_ with enhancing photoelectrochemical and photocatalytic performance. Appl. Catal. B Environ..

[32] Regmi C, Kshetri YK, Kim TH, Pandey RP, Lee SW (2017). Visible-light-induced Fe-doped BiVO_4_ photocatalyst for contaminated water treatment. Mol. Catal..

[33] Chen F (2016). Photo-reduction of bromate in drinking water by metallic Ag and reduced graphene oxide (RGO) jointly modified BiVO_4_ under visible light irradiation. Water. Res..

[34] Song S, Cheng B, Wu N, Meng A, Cao S, Yu J (2016). Structure effect of graphene on the photocatalytic performance of plasmonic Ag/Ag_2_CO_3_-rGO for photocatalytic elimination of pollutants. Appl. Catal. B Environ..

[35] Wu Z (2021). Development of a rGO-BiVO_4_ heterojunction humidity sensor with boosted performance. ACS Appl. Mater. Interfaces..

[36] Seid-Mohammadi A, Ghorbanian Z, Asgari G, Dargahi A (2019). Degradation of CEX antibiotic from aqueous environment by US/S_2_O_8_^2-^/NiO process: optimization using Taguchi method and kinetic studies. Desal. Wat. Treat..

[37] Seidmohammadi A, Vaziri Y, Dargahi A, Nasab H (2021). Improved degradation of metronidazole in a heterogeneous photo-Fenton oxidation system with PAC/Fe_3_O_4_ magnetic catalyst: biodegradability, catalyst specifications, process optimization, and degradation pathway. Biomass Convers. Biorefin..

[38] Antonopoulou M, Chondrodimou I, Bairamis F, Giannakas A, Konstantinou I (2017). Photocatalytic reduction of Cr (VI) by char/TiO_2_ composite photocatalyst: optimization and modeling using the response surface methodology (RSM). Environ. Sci. Poll. Res..

[39] Xu L, Wang J (2011). A heterogeneous Fenton-like system with nanoparticulate zero-valent iron for removal of 4-chloro-3-methyl phenol. J. Hazar. Mater..

[40] McQuaid HN, Mariotti D, Maguire PD (2023). Generation and delivery of free hydroxyl radicals using a remote plasma. Plasma Sources Sci. Technol..

[41] Hu X, Wang B (2021). Removal of pefloxacin from wastewater by dielectric barrier discharge plasma: Mechanism and degradation pathways. J. Environ. Chem. Eng..

[42] Chandana L, Reddy PMK, Subrahmanyam C (2015). Atmospheric pressure non-thermal plasma jet for the degradation of methylene blue in aqueous medium. Chem. Eng. J..

[43] Gaur N, Narasimhulu K, PydiSetty Y (2018). Recent advances in the bio-remediation of persistent organic pollutants and its effect on environment. J. Clean. Prod..

[44] Cubas ALV (2019). Effect of chemical species generated by different geometries of air and argon non-thermal plasma reactors on bacteria inactivation in water. Sep. Purif. Tech..

[45] Ahmadi E (2020). Synergistic effects of α-Fe_2_O_3_-TiO_2_ and Na_2_S_2_O_8_ on the performance of a non-thermal plasma reactor as a novel catalytic oxidation process for dimethyl phthalate degradation. Sep. Purif. Technol..

[46] Yi Z (2022). Decontamination of aniline and malathion on material surface by array cold atmospheric pressure plasma jet: Mechanism and decontamination pathways. J. Environ. Chem. Eng..

[47] Xu Z, Yu J (2011). Visible-light-induced photoelectrochemical behaviors of Fe-modified TiO_2_ nanotube arrays. Nanoscale..

[48] Zhang S, Wei Y, Metz J, He S, Alvarez PJ, Long M (2022). Persistent free radicals in biochar enhance superoxide-mediated Fe (III)/Fe (II) cycling and the efficacy of CaO_2_ Fenton-like treatment. J. Hazard. Mater..

[49] Chen G, Zhou M, Chen S, Chen W (2009). The different effects of oxygen and air DBD plasma byproducts on the degradation of methyl violet 5BN. J. Hazard. Mater..

[50] Magureanu M, Piroi D, Mandache NB, Parvulescu V (2008). Decomposition of methylene blue in water using a dielectric barrier discharge: Optimization of the operating parameters. J. Appl. phys..

[51] Chen Y (2022). Kinetic comparison of photocatalysis with H_2_O_2_-free photo-Fenton process on BiVO_4_ and the effective antibiotic degradation. Chem. Eng. J..

[52] Hu Y, Bai Y, Li X, Chen J (2013). Application of dielectric barrier discharge plasma for degradation and pathways of dimethoate in aqueous solution. Sep. Purif. Technol..

[53] Wang Y (2020). Fabrication of BiVO_4_/BiPO_4_/GO composite photocatalytic material for the visible light-driven degradation. J. Clean. Prod..

[54] Mahlalela LC (2020). Photocatalytic degradation of atrazine in aqueous solution using hyperbranched polyethyleneimine templated morphologies of BiVO_4_ fused with Bi_2_O_3_. J. Environ. Chem. Eng..

[55] Zheng K, Sun Y, Gong S, Jiang G, Zheng X, Yu Z (2019). Degradation of sulfamethoxazole in aqueous solution by dielectric barrier discharge plasma combined with Bi_2_WO_6_-rMoS_2_ nanocomposite: mechanism and degradation pathway. Chemosph..

[56] Guan R, Yuan X, Wu Z, Jiang L, Li Y, Zeng G (2018). Principle and application of hydrogen peroxide based advanced oxidation processes in activated sludge treatment: A review. Chem. Eng. J..

[57] Wang T, Qu G, Sun Q, Liang D, Hu S (2015). Evaluation of the potential of p-nitrophenol degradation in dredged sediment by pulsed discharge plasma. Water. Res..

[58] Sharma G (2019). Fabrication and characterization of novel Fe^0^@ Guar gum-crosslinked-soya lecithin nanocomposite hydrogel for photocatalytic degradation of methyl violet dye. Sep. Purif. Technol..

[59] Isari AA, Payan A, Fattahi M, Jorfi Kakavandi B (2018). Photocatalytic degradation of Rhodamine B and Real Textile Wastewater using Fe-Doped TiO_2_ anchored on Reduced Graphene Oxide (Fe-TiO_2_/rGO): Characterization and feasibility, mechanism and pathway studies. Appl. Surf. Sci..

[60] Sadati H, Ayati B (2023). Using a promising biomass-based biochar in photocatalytic degradation: highly impressive performance of RHB/SnO_2_/Fe_3_O_4_ for elimination of AO7. Photochem. Photobiol. Sci..

[61] Ling H (2023). Surfactant-enhanced bioremediation of petroleum-contaminated soil and microbial community response: A field study. Chemosph..

[62] Samy M (2023). Heterogeneous activation of persulfate by a novel nano-magnetite/ZnO/activated carbon nanohybrid for carbofuran degradation: Toxicity assessment, water matrices, degradation mechanism and radical and non-radical pathways. Process. Saf. Environ. Prot..

[63] Rapti I, Kosma C, Albanis T, Konstatineu I (2023). Solar photocatalytic degradation of inherent pharmaceutical residues in real hospital WWTP effluents using titanium dioxide on a CPC pilot scale reactor. Catal. Today..

